# Biological Evaluation in Resistant Cancer Cells and Study of Mechanism of Action of Arylvinyl-1,2,4-Trioxanes

**DOI:** 10.3390/ph15030360

**Published:** 2022-03-16

**Authors:** Jerome P. L. Ng, Mohit K. Tiwari, Ali Adnan Nasim, Rui Long Zhang, Yuanqing Qu, Richa Sharma, Betty Yuen Kwan Law, Dharmendra K. Yadav, Sandeep Chaudhary, Paolo Coghi, Vincent Kam Wai Wong

**Affiliations:** 1 Neher’s Biophysics Laboratory for Innovative Drug Discovery, State Key Laboratory of Quality Research in Chinese Medicine, Macau University of Science and Technology, Macau 999078, China; plng@must.edu.mo (J.P.L.N.); aanasim@gmail.com (A.A.N.); yeyutingfengmo@gmail.com (R.L.Z.); brookqu@gmail.com (Y.Q.); yklaw@must.edu.mo (B.Y.K.L.); 2 Laboratory of Organic and Medicinal Chemistry, Department of Chemistry, Malaviya National Institute of Technology, Jawaharlal Nehru Marg, Jaipur 302017, India; tiwari.mohitpharma22@gmail.com (M.K.T.); richasharmachem1@gmail.com (R.S.); 3 College of Pharmacy, Gachon University of Medicine and Science, Incheon City 21924, Korea; 4 Laboratory of Organic and Medicinal Chemistry (OMC Lab), National Institute of Pharmaceutical Education and Research (NIPER-R) Raebareli, Lucknow 226002, India; 5 School of Pharmacy, Macau University of Science and Technology, Macau 999078, China

**Keywords:** 1,2,4-trioxanes, P-glycoprotein, anticancer, molecular docking, mechanism of action

## Abstract

1,2,4-trioxane is a pharmacophore, which possesses a wide spectrum of biological activities, including anticancer effects. In this study, the cytotoxic effect and anticancer mechanism of action of a set of 10 selected peroxides were investigated on five phenotypically different cancer cell lines (A549, A2780, HCT8, MCF7, and SGC7901) and their corresponding drug-resistant cancer cell lines. Among all peroxides, only **7** and **8** showed a better P-glycoprotein (P-gp) inhibitory effect at a concentration of 100 nM. These in vitro results were further validated by in silico docking and molecular dynamic (MD) studies, where compounds **7** and **8** exhibited docking scores of −7.089 and −8.196 kcal/mol, respectively, and remained generally stable in 100 ns during MD simulation. Further experiments revealed that peroxides **7** and **8** showed no significant effect on ROS accumulations and caspase-3 activity in A549 cells. Peroxides **7** and **8** were also found to decrease cell membrane potential. In addition, peroxides **7** and 8 were demonstrated to oxidize a flavin cofactor, possibly elucidating its mechanism of action. In conclusion, apoptosis induced by 1,2,4-trioxane was shown to undergo via a ROS- and caspase-3-independent pathway with hyperpolarization of cell membrane potential.

## 1. Introduction

Cancer is one of the leading causes of death globally, responsible for an estimated 9.6 million deaths in 2020 [1]. Lung cancer remains the most frequently occurring cancer, with an estimated 1.8 million deaths in 2020 [2]. Chemotherapy is a conventional remedy for cancer. However, this approach is always restricted by drug efficacy, high toxicity, and emerging development of drug resistance.

**Figure 1:**
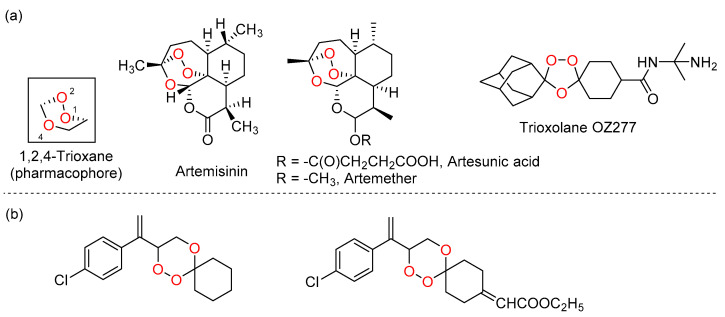
(**a**) Structures of artemisinin and its derivatives; (**b**) structures of synthetic halogenated arylvinyl-1,2,4-trioxanes as antimalarial agents.

Artemisinin (ART), also known as qinghaosu, is a sesquiterpene lactone endoperoxide that contains 1,2,4-trioxane as the pharmacophore and is abundant in the hexane extract of a Chinese medicinal plant, *Artemisia annua* (Figure 1a). ART and its semisynthetic derivatives, artesunic acid and dihydroartemisinin (Figure 1a), not only exhibit activity against malaria and even drug-resistant *Plasmodium* but also display inhibitory activity toward other diseases, including cancer in vitro and in vivo [3,4,5].

Understanding of the active pharmacophore 1,2,4-trioxane ring and the lipophilicity for drug efficacy [6,7,8], and the poor extraction yield of artemisinin from *Artemisia annua* have facilitated the development of novel biologically active halogen-substituted synthetic 1,2,4-trioxanes (Figure 1b) [9,10]. The pharmacological activities of these 1,2,4-trioxanes have also been suggested to modulate oxidative stress via reactive oxygen species (ROS) or carbon-centered radicals generated by the cleavage of endoperoxide bridges [11]. Moreover, a mechanistic model of endoperoxides has been previously reported, whereby endoperoxides mediate their activity through interaction with cofactors of redox-active flavoenzyme [12].

**Figure 2:**
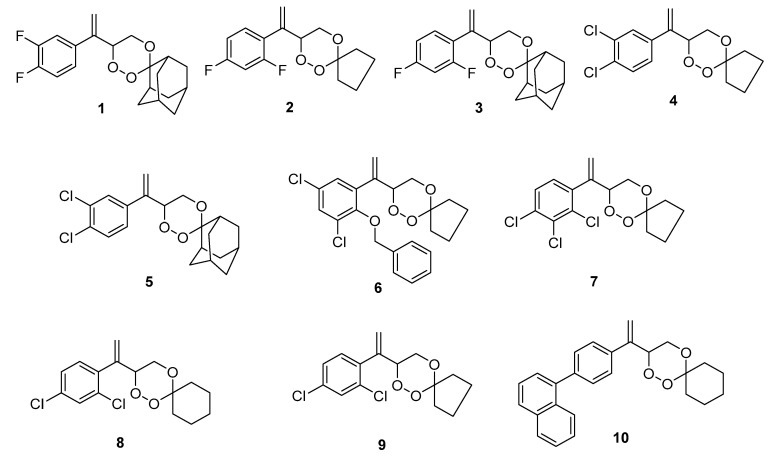
Structures of 1,2,4-trioxanes **1**–**10** prepared for this study.

Recently, we have reported some novel halogenated and non-halogenated arylvinyl-1,2,4-trioxanes with activities against chloroquine-resistant Pf INDO strain of *P. falciparum* and lung cancer A549 cell lines [13,14]. In this study, in-depth investigations were carried out on in vitro anticancer activities of the previous series of 1,2,4-trioxanes (Figure 2), as well as the mechanism of cytotoxic action. These compounds were first examined on five cancer cell lines (A549, A2780, HCT8, MCF7, SGC7901) and the corresponding drug-resistant cancer cell lines. All compounds were also tested for P-gp inhibition in vitro [15], and the P-gp inhibitory effect was further validated by the docking studies and molecular dynamics. Selected compounds were further subjected to analysis of ROS production and cell membrane potential [16]. Furthermore, the activation of caspase-3, which is the hallmark of apoptotic cell death, and the reactivity with flavine were studied.

## 2. Results and Discussion

### 2.1. Chemistry

Ten arylvinyl-1,2,4-trioxanes (Figure 2) were prepared by using the typical photooxygenation protocol of endoperoxide synthesis as described in the literature [17].

### 2.2. Biological Studies

#### 2.2.1. Cytotoxicity Studies

The cytotoxicity of peroxides **1**–**10** was examined on a series of wild-type cancer cells (human lung, ovarian, breast, colorectal, and gastric cancer cell lines: A549, A2780, MCF7, HCT8, and SGC7901) and the corresponding drug-resistant cancer cell lines (A549T, A2780/CDDP, MCF7/ADR, HCT-8/T, and SGC7901/CDDP). Two widely used anticancer agents, paclitaxel (PTX) and cisplatin (CDDP), were used as reference drugs. Previously, peroxide **7** was reported to be the most active compound against A549 cancer cells among the series of 1,2,4-trioxanes, showing approximately 145-fold more cytotoxic along with higher selectivity in comparison to the reference drug artemisinin [13]. In addition, peroxide **8** also showed potent in vitro anticancer activity with IC_50_ < 1 μΜ [13].

Ten selected compounds were previously tested against lung cell lines A549 and BEAS-2B [13]. Compounds **7** and **8** were further assessed against LO2 and CCD-19Lu normal cells (Table S1 and Figure S10 Supplementary Materials). Compared to CDDP and PTX, both **7** and **8** showed higher in vitro activity against different drug-resistant cancer cell lines (Table S1 and Figures S1–S9 Supplementary Materials), where they exhibited better selectivity index (MCF7/MCF7/ADR) in breast cancer cells (S.I. = 5.24 for **7**; S.I. = 3.13 for **8**).

All cyclic peroxides except compound **2** showed better activity for lung A549T cancer cell lines compared to PTX and CPT with IC_50_ < 5 μΜ (Table 1 and Figure S1 Supplementary Materials). Of note, compound **7** was the most potent among the series with IC_50_ of 0.69 μM against lung cancer cells A549 [13].

#### 2.2.2. Rhodamine 123 Exclusion Assay

The ATP-binding cassette (ABC) transporter is a transmembrane protein that can be found in almost all organisms. Its overexpression, in particular P-glycoprotein (P-gp/ABCBl), has been known to facilitate multidrug resistance in cancer cells, which efflux chemotherapeutic drugs to escape from cell death [18]. Recently, we found that some ozonides exhibited inhibition against ABCB5 overexpression in HepG2 cancer cells. In this regard, the inhibition of ABCB5 can counteract the multidrug resistance in liver cancer [19], proposing that peroxide compounds may inhibit ABCB1 as well.

**Figure 3:**
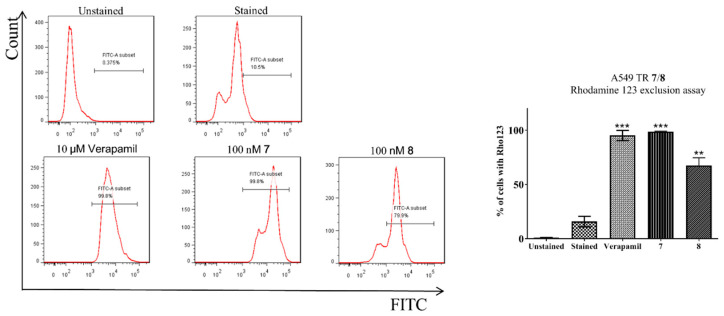
The intracellular level of rhodamine 123 accumulation in taxol-resistant A549 cells acquired by flow cytometry analysis. Cells were treated with 5 μM of rhodamine 123 for 1 h at 37 °C along with 10 µM of verapamil (positive control), 100 nM of **7** or **8**. Control sample (arbitrarily set as 100%) was acquired without any compound treatment. Data are expressed as % of rhodamine 123 accumulation (** *p* < 0.01, *** *p* < 0.001).

In order to examine the inhibitory effect of compounds **1**–**10** on ABCB1 activity, a rhodamine 123 (Rho123) exclusion assay was employed to measure the intracellular level of Rho123 accumulation in taxol-resistant A549 cancer cells in response to drug treatment (Figure 1 and Figures S11–S18 in Supplementary Materials). As shown in Figure 3, the Rho123 accumulation reached ~11% in A549T cells without drug treatment (control). Upon treatment of 10 μM of verapamil (positive control), Rho123 accumulation was elevated to 99.8%, indicating the suppression of ABCB1 activity. High accumulation of Rho123 was also observed for compounds **7** and **8** at an even lower concentration (100 nM) among the trioxane series. Thus, it suggests that trioxanes **7** and **8** strongly inhibited ABCB1 in A549T cancer cells.

However, the cytotoxicity of **7** and **8** against A549T cells was decreased 4 and 14 times, respectively, in comparison with that against A549 cells (Table 1). Thus, the results evidence that the resistance to **7** and **8** is independent of the role of P-gp.

Given the promising IC_50_ values of **7** and **8** against A549 cells [13] and the outstanding P-gp inhibitory effect among the series, these two promising compounds were subjected to further analyses of ROS production, caspase-3 activity, and cell membrane potential.

#### 2.2.3. Measurement of ROS Generation

ROS is highly associated with cell growth and apoptosis, and its production has been suggested to participate in the mechanistic pathway of ROS-dependent apoptosis [20]. ROS exhibits diverse cellular functions, including the modulations of cell proliferation and migration, induction of apoptosis, and immune regulation. ROS production during inflammation can cause DNA damage and, subsequently, promote or even accelerate carcinogenesis. Moreover, ROS was found to implicate the apoptotic pathway of lung cancer cells such as lung adenocarcinoma A549 cells [21,22]. The antimalarial and anticancer activities of ART is commonly arising from the generation of free radicals through the breakage of the endoperoxide bridge [23,24]. The generated free radicals then disturb the redox equilibria in cells and therefore induce a ROS increase by various redox enzymes [25].

Our preliminary results demonstrated that endoperoxides interacted with cofactors to enhance the consumption of NADPH and the generation of ROS, leading to their antimalarial activity [12]. Of note, iron is not considered to be essential as suggested previously, but it certainly will play a role in modulating oxidative stress [25].

**Figure 4:**
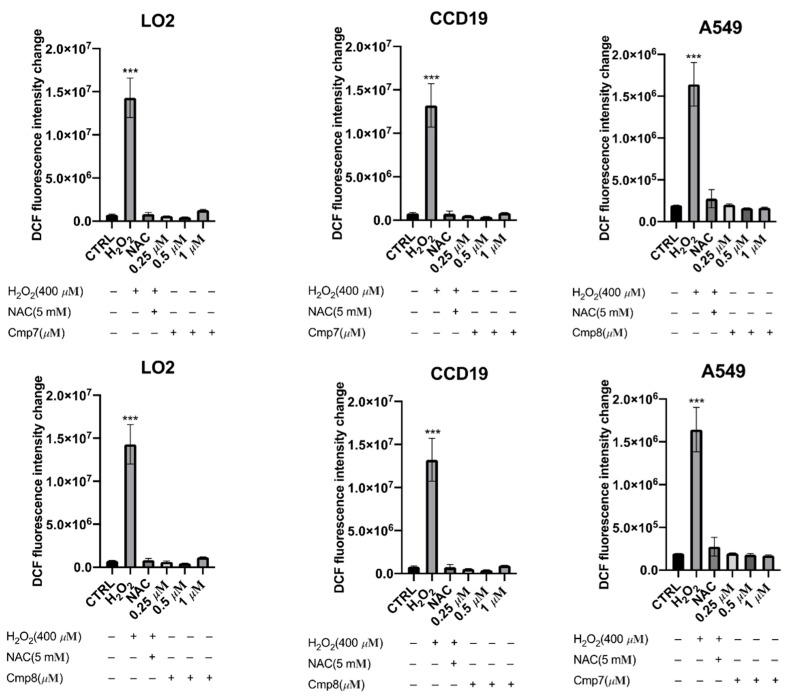
ROS generation for compounds **7** and **8** in cancer cells (A549) compared to normal cells (CCD-19Lu and LO2). Intracellular ROS generation was quantified by using DCF dye and measured by the relevant fluorescent signal. By *t*-test analysis, *** *p* < 0.001, comparing to the control group.

To explore the role of ROS in the endoperoxide-induced cell death, the ROS generation induced by compounds in normal (LO2 and CCD-19Lu) and cancer (A549) cells was assessed by measuring DCF fluorescence using a microplate reader. Treatment of cells with hydrogen peroxide (H_2_O_2_), a positive control, showed a dramatic increase in ROS accumulation (Figure 4). When the cells were co-treated with NAC, a ROS scavenger, ROS generations in cells by H_2_O_2_ were inhibited, showing its role in the examination of ROS inducers.

According to the previous results, the most potent compounds **7** and **8** against A549 cancer cells were selected for the examination of ROS generation. Results showed that no ROS accumulation was observed in all cell lines (LO2, CCD19, and A549) treated with compounds **7** and **8** with a concentration up to 1 µM (Figure 4). This demonstrated that endoperoxide-induced cell death was not related to ROS generation.

Notably, ART that also possessed an endoperoxide structure showed an increase in ROS in liver cancer and breast cancer cells [26,27]; however, further studies proved ROS not involved in the mechanism of endoperoxide-induced apoptosis. Similarly, our results concluded that endoperoxides **7** and **8** induce ROS-independent cytotoxicity in A549 cells.

#### 2.2.4. Measurement of Cell Membrane Potential

A homeostatic balance of cell membrane potential, also known as plasma membrane potential, is vital for cell survival. The cell membrane potential is associated with the balance of intracellular and extracellular ions, which are typically regulated by ion channels and pumps. Several studies have shown that a loss of intracellular ions promotes early apoptotic events leading to cell death [28,29].

**Figure 5:**
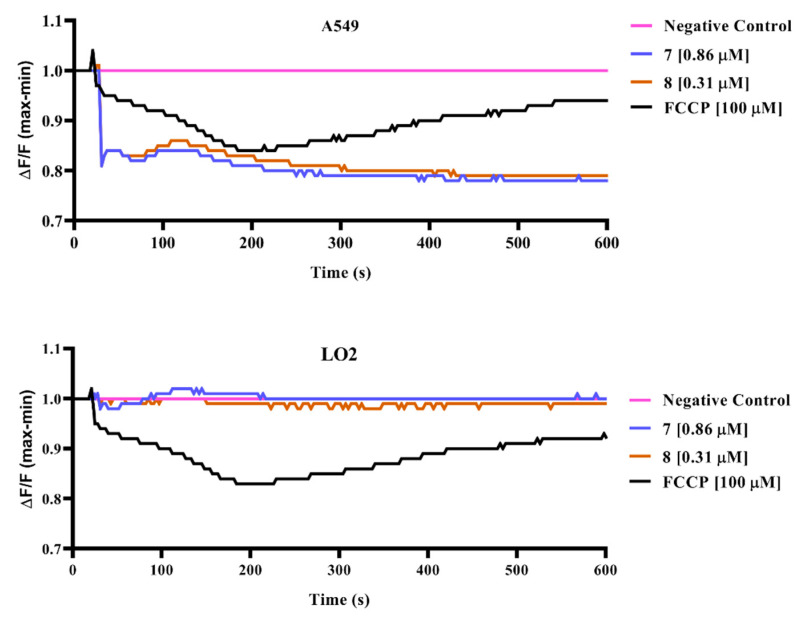
The effects of compound**s 7** and **8** on cell membrane potential. The alternation of cell membrane potential was measured in terms of fluorescence. An increase in fluorescence intensity indicates depolarization of cell membrane potential, while a decrease in fluorescence intensity indicates hyperpolarization of cell membrane potential.

To examine the effects of selected peroxides, **7** and **8**, on cell membrane potential, variations of cell membrane potential (ΔF/F) in LO2 and A549 cells were determined by FLIPR Membrane Potential Assay Kit (Figure 5). Control treatment with carbonyl cyanide p-trifluoromethoxyphenylhydrazone (FCCP) decreased the intracellular fluorescence intensity, denoting hyperpolarization of cell membrane potential. Similarly, both peroxides induced a time-dependent decrease in cell membrane potential in A549 cancer cells, indicating that hyperpolarization of cell membrane potential played a crucial role in endoperoxide-induced apoptosis. Meanwhile, treatments with peroxides in normal LO2 cells showed no significant influence on cell membrane potential. This implied the selectivity of endoperoxides **7** and **8** toward A549 cancer cells to induce apoptotic pathways by hyperpolarization of cell membrane potential.

#### 2.2.5. Caspase-3 Activity Assay

Caspases belong to a family of protease enzymes that are commonly found to be involved in apoptosis. They are typically activated in a protease cascade, resulting in the degradation of cellular components [30]. In particular, caspase-3 is frequently activated in the apoptotic process, and some of its exerted effects have been reported to be crucial for cell death [31]. Moreover, a change in intracellular ions was found to influence caspase-3 activity in apoptosis [28].

**Figure 6:**
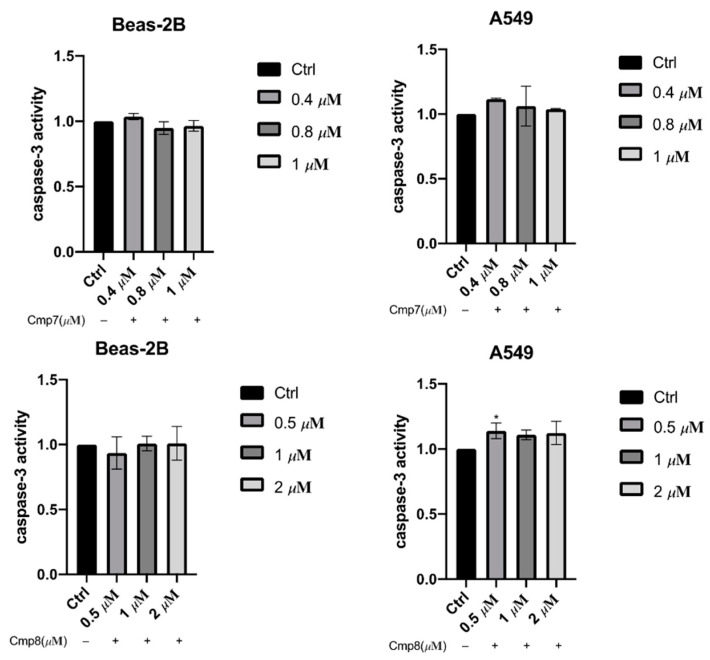
Fluorometric determination of caspase-3 activation for endoperoxides **7** and **8** using Ac-DEVD-pNA. The caspase-3 activation index of the control group was set as 1.0. Data points for caspase-3 activity represent the means of triplicate determinations ± S.D. By t-test analysis, * *p* < 0.05, compared to the control group.

In view of the hyperpolarization of membrane potential induced by **7** and **8**, caspase-3 activity downstream of the apoptotic pathway was investigated. The fluorogenic substrate Ac-DEVD-pNA was employed to elucidate whether endoperoxides **7** and **8** induced activation of caspase-3 for cell death. For cancer cells A549, the caspase-3 activity was slightly increased by both endoperoxides but was independent of endoperoxide concentration (Figure 6). This implied that activation of caspase-3 may be involved in the apoptosis; however, caspase-3 activation is not critical for endoperoxide-induced cell death. Notably, both compounds showed no significant effects on caspase-3 activity in normal cells BEAS-2B at the same drug concentrations used in A549 cells.

#### 2.2.6. Reactivity Studies Using Dihydroflavins and Leucomethylene Blue

In our previous studies, ART and its semisynthetic derivatives, which contain 1,2,4-trioxane core, were demonstrated to exhibit antimalarial effect by oxidizing dihydroflavin (RFH2), cofactors of redox-active flavoenzyme [12]. The radicals generated by endoperoxides disrupt the redox homeostasis and subsequently lead to the death of parasites. Moreover, ART was also reported to be activated by iron(II)-heme [32]. Other than antimalarial activity, some bridged ozonides were also found to show promising and selective cytotoxicity toward liver cancer cell lines [33].

Similar to this study, Singh et al. studied trioxanes and their reactions with different iron(II) salts or a combination of hemin and reduced glutathione under aerobic and anaerobic conditions to elucidate the mechanism of action [34].

**Figure 7:**
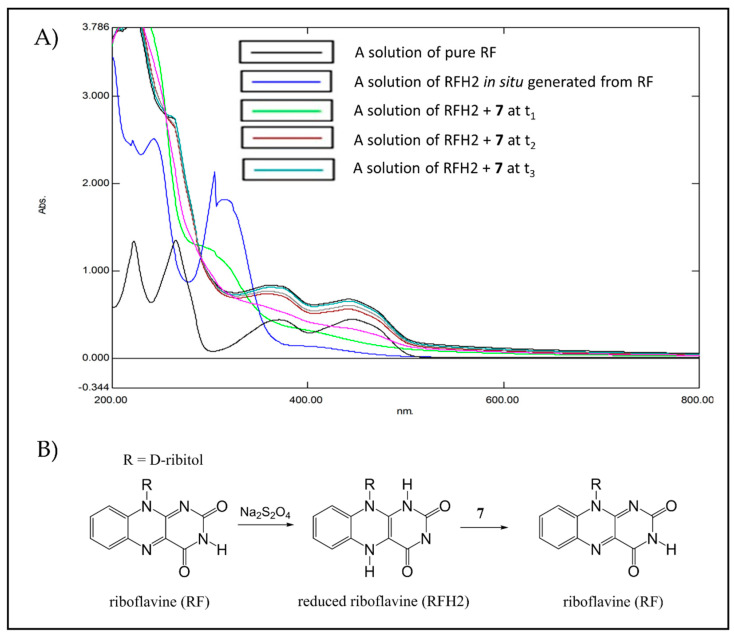
(**A**) UV-Vis analysis of oxidation of reduced riboflavin (RFH2) by trioxane **7**. RF (6.6 × 10^−^^4^ mmol) was completely converted to RFH2 (disappearance of absorption bands at 370 and 445 nm) by the treatment with sodium dithionite (1.4 × 10^−^^4^ mmol) under argon at 37 °C. Trioxane 7 (4 × 10^−^^4^ mmol) in MeCN was added, and the absorptions of the mixture at 370 and 445 nm were monitored at an interval (t_1_–t_3_) of 20 s until complete oxidation of RFH2 back to RF (within 10 min). (**B**) Proposed mechanism of the in situ generation of RFH2 from RF and the oxidation of RFH2 by **7** to regenerate RF.

**Figure 8:**
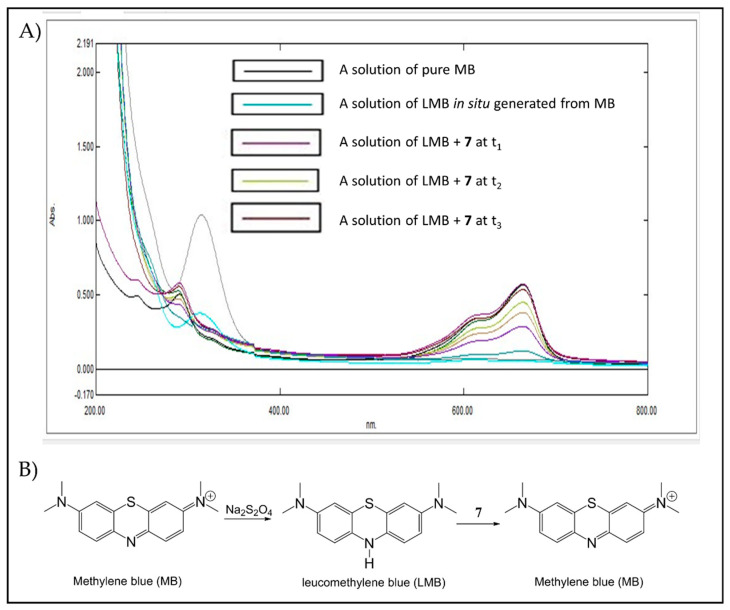
(**A**) UV-Vis analysis of oxidation of leucomethylene blue (LMB) by trioxane **7**. MB (6.6 × 10^−^^4^ mmol) was completely converted to LMB (disappearance of the absorption band at 650 nm, azure line) by the treatment with sodium dithionite (1.4 × 10^−^^4^ mmol) under argon at 37 °C. Trioxane 7 (4 × 10^−^^4^ mmol) in MeCN was added, and the absorption of the mixture at 650 nm was monitored at an interval (t_1_–t_3_) of 20 s until complete oxidation of LMB back to MB (within 10 min). (**B**) Proposed mechanism of the in situ generation of LMB from MB and the oxidation of LMB by **7** to regenerate LB.

UV-Vis spectrometry was used to monitor the reactivity of trioxanes by oxidizing RFH2 (flavin cofactor) and leucomethylene blue (LMB). As shown in Figure 7A,B RFH2 that was generated from pretreatment of riboflavin (RF) with sodium dithionite exhibited two absorption bands at 370 and 445 nm. Upon addition of endoperoxide **7**, these two absorption bands disappeared over time due to rapid oxidation of RFH2 back to RF. Similar observations were also observed for the reaction of RFH2 with endoperoxide **8** (Figure S19, Supplementary Materials). Meanwhile, pretreatment of methylene blue (MB) with sodium dithionite afforded LMB, which exhibited an absorption band at 650 nm (Figure 8A,B). Similarly, the disappearance of this absorption band is attributed to the oxidation of LMB to MB by trioxane **7**. Of note, sodium dithionite alone has no effect on trioxane under these conditions.

Apart from the redox reaction of endoperoxide with iron (II) as reported in the literature [35], our results revealed that such reaction could also be achieved by flavin cofactor, thus demonstrating the apoptotic pathway induced by **7** and **8**.

The multidrug resistance in cancer cells is partly attributed to the overexpression of the adenosine triphosphate (ATP)-driven transmembrane efflux pumps. ATP takes an irreplaceable role in the functions of efflux pumps/transporters or phosphorylations. Hence, drug resistance in cancer may be addressed by inhibiting the production of ATP in cancer cells or by modulating the catalytic cycle of ATP hydrolysis [36]. In the synthesis of ATP, ATP synthase is driven by a concentration gradient of protons in mitochondria. The proton gradient is generated by an electron transport chain with electrons donated mainly from cofactors (NADH, FADH2). The nature of the electron transport chain originates from various oxidation-reduction reactions. Therefore, one possible way to inhibit the production of ATP is to consume FADH_2_ and NADH. In this way, we proposed that apoptosis could be promoted by P-gp inhibitors trioxanes **7** and **8**, similar to other reported peroxides [19].

### 2.3. In Silico Studies

#### 2.3.1. Molecular Docking of P-gp for **7** and **8**

As demonstrated in Section 2.2.2, peroxides **7** and **8** among all tested compounds showed the best P-gp inhibitory effect in vitro. Their P-gp inhibitory effects were further validated by molecular docking.

**Figure 9:**
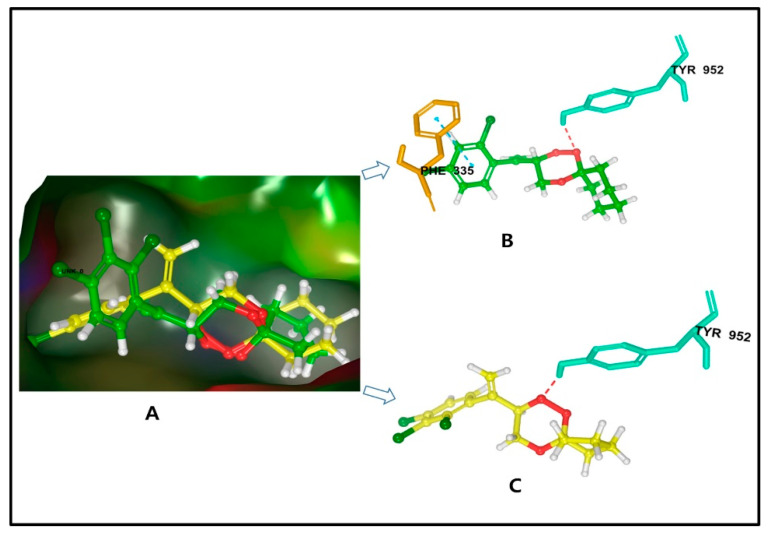
(**A**) Superposition of docked pose; the binding site amino acid residues interactions within 3Å is shown only for (**B**) **7** and (**C**) **8**.

The docking results for compound **7** exhibited a suitable binding with P-gp, showing a docking score of −7.089 kcal/mol. Detailed analysis revealed that it formed a halogen bond of length 1.8Å to the aromatic residue that was, tyrosine-952. The chemical nature of binding site residues in docking pose, was hydrophobic, for example, Leu-64, Leu-338 (leucine); Ile-305, Ile-339 (isoleucine); Met-67, Met-68, Met-985, Met-948 (methionine); and aromatic (hydrophobic), for example, Phe-727, Phe-982 (phenylalanine), Tyr-949, Tyr-952, Tyr-309 (tyrosine). Thus, the bound complex showed strong hydrophobic interactions with P-gp, resulting in a relatively stable complex (Figure 9a).

Similarly, the docking results for **8** against P-gp showed a suitable docking score of −8.196 kcal/mol and formed a halogen bond of length 2.2Å to the aromatic residue that was, tyrosine-952 along with π-π stacking with phenylalanine-335. In the docking pose of the ligand complex, the binding site residues within a radius of 3Å were hydrophobic, for example, Leu-64, Leu-338 (leucine); Ile-339 (isoleucine); Met-67, Met-68, Met-985, Met-948 (methionine); aromatic (hydrophobic), for example, Phe-982 (phenylalanine), Tyr-949, Tyr-952, Tyr-309 (tyrosine); and Gln-945 (glutamine). Thus, the bound complex showed a strong hydrophobic interaction and binding stability with the target protein (Figure 9b).

The docking studies confirmed that the studied compounds, **7** and **8**, represent a valid candidate as the potential P-gp inhibitor. Finally, the control molecule artesunate showed the docking score of −6.252 kcal/mol (Table S2 Supplementary Materials) and showed π-π stacking with phenylalanine-982. In addition, the docking results are also validated by using cocrystal ligand (zosuquidar), demonstrating an RMSD value of 0.3312 Å as described in Figure S20 of Supplementary Materials. Hence, the molecular simulation procedure in reproducing the experimental binding score is reliable and is predicted as true positive.

#### 2.3.2. Molecular Dynamics Simulation of **7** and **8**

**Figure 10:**
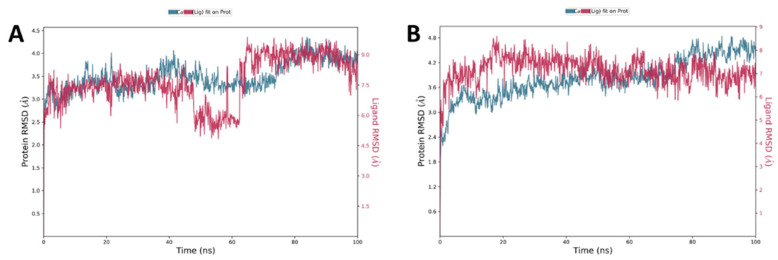
Time-dependent root mean square deviation (RMSD) (Å) for **7** (**A**) and **8** (**B**) during the 100 ns molecular dynamics simulation.

Many studies have used the MD simulation approach to investigate the binding of ligand with protein. All-atom MD simulations were carried out for docked complex of *P-gp-ligand (***7** and **8**) and compared with the free state of *P-gp*. Plots for RMSD, RMSF, Rg, and SASA fluctuations along with hydrogen bonds analysis were generated and analyzed in detail. The analysis of RMSD assists in understanding the stability of a protein and protein-ligand complex. For the exploration of structural deviations in HSA, we used RMSD examination of the simulated protein and protein-ligand complex. As examined, for *P-gp* and *P-gp-ligand **(*****7** and **8**) complex, both structures reached the equilibrium phase without any major shift in the RMSD pattern. The protein-ligand complex was stable throughout the simulation of 100 ns. The analysis of the generated plots suggested that there was a little fluctuation at the beginning in the RMSD values of the *P-gp-ligand (***7** and **8**), possibly due to the initial adjustment of the system. As a whole, *P-gp* showed a small number of fluctuations in its backbone in the simulation, but no significant shift was observed (Figure 10).

**Figure 11:**
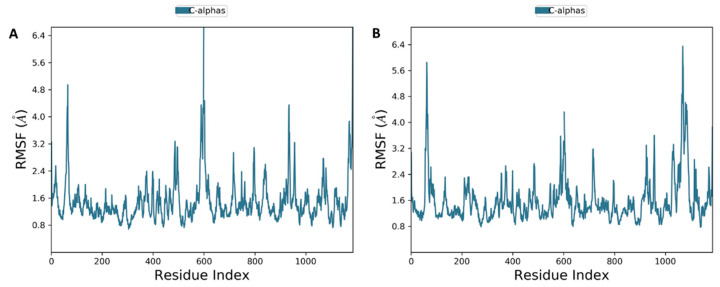
Root mean square fluctuations of the Cα atom during the 100 ns molecular dynamic simulation. The ligand contact with protein residues is marked with blue-colored vertical bars compound **7** (**A**) and **8** (**B**).

The result of MD of both compounds (**7** and **8**) bound to the P-gp protein showed that the stability of docking simulations complex structures is retained in the binding pocket throughout the simulations (Figure 10). Cα atoms of the protein backbone were fixed by fixing translational and rotational spinning to the corresponding initial structure for MD run during the RMSD calculations of the complex protein. The RMSD plots for **7** and **8** showed an overview of protein conformational perturbation during the binding (Figure 10). The RMSD analysis showed a slightly higher RMSD for **8** in comparison with **7**, but insignificant deviation was observed with respect to the actual P-gp binding site protein backbone deviation. The trajectory patterns of the P-gp with either compound **7** or **8** were quite similar during the entire period of MD simulations (100 ns). Thus, it states that the reference structure of the P-gp-ligand complex remained unchanged after binding with the tested compounds. In addition, no significant conformational changes were observed for all compounds before and after bindings. The RMSD plot showed that, after 5 ns, the **7** and **8** systems attained the equilibrium and then oscillated further with an RMSD of 2.5 Å and 2.4 Å, respectively. The backbone atoms motions and local changes in secondary structure elements were analyzed to investigate the fluctuations in the residues of P-gp. As shown in the RMSF plot (Figure 11A,B), despite both compounds **7** and **8** are fall in the same binding site amino acid residues, only the complex system of **7** showed relatively larger backbone residues fluctuations.

**Figure 12:**
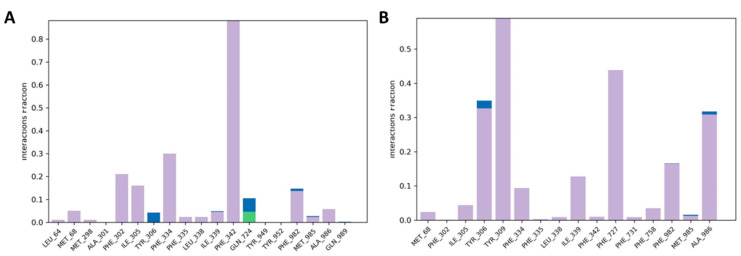
Protein-ligand contacts histogram and ligand interaction of compounds **7** (**A**) and **8** (**B**).

Hydrogen bonds are necessary for maintaining the structural conformation of a protein. Analysis of hydrogen bonding is useful to assess the stability of a protein and protein-ligand complex. The stability of halogen bond has been calculated between all possible donors and acceptors into the active site domain of the P-gp protein; and the formations of halogen and hydrogen bonds between the active site residues and compounds are almost maintained during the whole scale run of MD trajectories (Figure 12A,B). Both compounds maintained one halogen and H-bond bonds within the active site’s domain. Along with the stable hydrogen bond formation, the RMSD of these compounds had maintained because of the binding of compounds with active amino acids located on the mostly loop region. The simulation revealed that the halogen, H-bond, or π-π stacking have been formed with the receptor and show a suitable dynamic simulation trajectory. Molecular dynamics simulation trajectory clearly revealed that the binding poses of both compounds retain the H-bond with backbone atom of Tyr-952 residue throughout the whole 100 ns dynamics trajectories as shown in Figure 12A,B.

Finally, the average free binding energy of both compounds (**7** and **8**) along with artesunate were calculated, and the result was shown in Table S3 of Supplementary Materials. The average binding free energy for **7_,_ 8,** and artesunic acid were −96.714, −93.841, and −70.435 kcal/mol, respectively. The binding affinity in the form of docking score values of compounds were −8.196, −7.089, and −6.252 kcal/mol, which is satisfactory with the measured free energy of binding. Based on the RMSD, RMSF, and MM-GBSA results showed that these selected compounds were found to be stable, and the P-gp protein-ligands conformation remains unchanged. This further validated the in vitro results that compounds **7** and **8** exhibited an outstanding P-gp inhibitory activity at nanomolar concentration.

## 3. Materials and Methods

### 3.1. General

Human normal hepatocytes, LO2, lung epithelial cells, BEAS-2B, and human normal fibroblasts, CCD19Lu, as well as human cancer (A549, A2780, MCF7, HCT8, and SGC7901) and drug-resistant cancer cells (A549T, A2780/CDDP, MCF7/ADR, HCT-8/T, and SGC7901/CDDP) were all purchased from ATCC (Manassas, VA, USA). Cells were cultured in RPMI-1640 medium consisting of 10% fetal bovine serum and antibiotics penicillin (50 U/mL) and streptomycin (50 μg/mL; Invitrogen, Paisley, U.K.), and were incubated at 37 °C in a 5% humidified CO_2_ incubator).

### 3.2. Chemistry

All chemicals were purchased from Sigma-Aldrich (Shanghai, China) and were used directly unless otherwise specified. All 1,2,4-trioxanes used in this study were prepared as same as we reported previously [13] using known synthetic protocols in literature [17]. All compound characterizations were available in our previous publication [13].

### 3.3. Biological Screening Studies

#### 3.3.1. Cytotoxicity Drug Assay

Stock solutions of all test compounds were prepared by dissolving compounds in DMSO at a concentration of 100 mM and were stored at −20 °C before use. The 3-(4,5-dimethylthiazole-2yl)-2,5-diphenyltetrazolium bromide (MTT) assay was employed for assessing cytotoxicity. Briefly, normal/cancer cells with a density of 4 × 10^3^ cells/well were seeded in 96-well plates overnight, followed by treatment with gradient concentrations of selected compounds (0.19–100 μM) for 72 h. Cells without any compound treatment were used as a control. After that, 10 μL of MTT solution (5 mg/mL) was added to each well, and the cells were further incubated at 37 °C for 4 h followed by the addition of 100 μL of solubilization buffer (10 mM HCl in 10% SDS) and cultured overnight. Absorbance (A) of each well was then determined at 570 nm. The cytotoxicity was expressed in cell viability (%) calculated by using the following formula: Cell viability (%) = Absorbance (treated)/Absorbance (control) × 100%. Representative graphs of at least three independent experiments were shown in Figures S1–S10 of Supplementary Materials.

#### 3.3.2. Rhodamine 123 Exclusion Assay

Taxol-resistant A549 (A549T) cells were seeded in 6 well plates with a density of 2 × 10^5^ cells/well and incubated at 37 °C for 24 h in a 5% CO_2_ incubator. The cells were incubated with or without 10 μM of verapamil (positive control) and compounds **1**–**10** for 6 h or 24 h at 37 °C. Subsequently, rhodamine 123 (5 μg mL^−1^, Rho123) was added to each well followed by incubation for another 1 h at 37 °C. The cells were then washed with ice-cold PBS five times and resuspended in 400 μL of PBS before flow cytometry analysis. Intracellular accumulation of Rho123 was determined by measuring the fluorescence using a flow cytometer at an excitation wavelength of 488 nm and an emission wavelength of 525 nm. All data acquisition and analysis were performed with CellQuest (BD Biosciences, San Jose, CA, USA) with at least three independent experiments. Data for Rho123 accumulation were shown as the means of fluorescence intensity (control group was arbitrarily set as 100%).

#### 3.3.3. Detection of ROS Generation Assay

Cells were seeded in 96-well plates at a density of 1 × 10^4^ cells/well and cultured overnight. Before drug treatment, cells were pre-treated with dichlorodihydrofluorescein (DCF, 10 μM) and NAC (5 mM) for 1 h at 37 °C. After removal of the supernatant, cells were treated with 1× HBSS (control), H_2_O_2_ (400 μM in 1× HBSS, positive control), H_2_O_2_ (400 μM) + NAC (5 mM) and compounds **7**–**8** (specified concentration in 1× HBSS) respectively. After incubation for 30 min, fluorescence readings were acquired by an auto microplate reader using an excitation wavelength of 488 nm and emission wavelength of 530 nm. All experiments were performed with at least three independent experiments.

#### 3.3.4. Colorimetric Determination of Caspase-3 Activity

Caspase-3 activity was measured by using Caspase-3 Assay Kit, Colorimetric (Abbkine, Wuhan, China), according to the manufacturer’s instructions. Cells were seeded with a density of 1.0 × 10^5^ cells/well and cultured for 24 h. Cells were treated with a concentration gradient of compounds **7** and **8** for 24 h. Cells were collected and washed twice with cold PBS. Cells were lysed in cell lysis buffer provided in the kit. The supernatant of the extract was transferred to a 96-well plate with 50 μL/well. A total of 50 μL of 1× reaction buffer was added to each well, followed by the addition of 5 μL of 4 mM Ac-DEVD-pNA. After incubation at 37 °C for 60 min, caspase-3 activity was measured by using an auto microplate reader at 405 nm.

#### 3.3.5. Measurement of Cell Membrane Potential

Cell membrane potential was measured by FLIPR Membrane Potential Assay Kits (R8126, Molecular Devices, Sunnyvale, CA, USA) according to the manufacturer’s instructions. A total of 50,000–80,000 FLIPR membrane potential RED-stained cells/well were simultaneously treated on the FLIPR Tetra High-Throughput Cellular Screening System (Molecular Devices, Sunnyvale, CA, USA). Real-time membrane potential changes were monitored for 3600 s at 530 and 565 nm excitation and emission filters at 3 s reading intervals.

### 3.4. Reactivity Studies

#### 3.4.1. Oxidation of Reduced Riboflavin (RFH2) by Peroxide **7**

Riboflavin (RF, 5 mg) was added to a solution of degassed MeCN/pH 7.4 buffer (1:1, 2 mL). A solution of RF (6.6 × 10^−^^4^ mmol) in a UV cuvette was prepared by diluting 100 μL of the mixture with degassed water (900 μL). The cuvette was sealed with a septum immediately after the addition of solid sodium dithionite (1 mg, 1.4 × 10^−^^2^ mmol), and the mixture was stirred under nitrogen. Once the reduction of RF was completed as judged by the disappearance of absorptions at 445 and 370 nm, a solution of endoperoxide **7** or **8** (4 × 10^−^^4^ mmol) in MeCN (100 μL) was added to the cuvette by syringe. Absorptions at 370 and 445 nm were monitored by UV-Vis spectrometer at an interval of 20 s until complete oxidation had been performed (within 10 min).

#### 3.4.2. Oxidation of Reduced Leucomethylene (LMB) by Peroxide **7**

Methylene blue (MB, 5 mg) was added to a solution of degassed MeCN/pH 7.4 buffer (1:1, 2 mL). A solution of MB (6.6 × 10^−^^4^ mmol) in a UV cuvette was prepared by diluting 100 μL of the mixture with degassed water (900 μL). The cuvette was sealed with a septum immediately after the addition of solid sodium dithionite (1 mg, 1.4 × 10^−^^2^ mmol), and the mixture was stirred under nitrogen. Once the reduction of MB was completed as judged by the disappearance of absorption at 650 nm, a solution of endoperoxide **7** (4 × 10^−^^4^ mmol) in MeCN (100 μL) was added to the cuvette by syringe. Absorption at 650 nm was monitored by UV-Vis spectrometer at an interval of 20 s until complete oxidation had been performed (within 10 min).

### 3.5. In Silico Studies of Trioxanes with P-Glycoprotein Proteins

#### 3.5.1. Molecular Docking

The prepared files of the receptor and ligand structures were used for docking study performed by the maestro software. The X-ray crystal structure of P-glycoprotein (P-gp), a cancer target protein, was obtained from the Protein Data Bank (PDB: 6FN1). The protein preparation wizard of Schrodinger Suite was then used to prepare the protein structure for docking analysis. During protein preparation, all water molecules were removed, missing side chains and hydrogen atoms were added, and all-atom force field (OPSL3) charges and atom types were assigned accordingly. All compounds were prepared using the LigPrep module of the Schrödinger Suite, generating multiple conformers and Epik-based ionization states [19]. Detailed docking procedures were described by Tiwari et al. [13].

#### 3.5.2. Molecular Dynamics (MD) Simulations

Molecular dynamics (MD) simulations were performed by Desmond version 5.3 with an inbuilt OPLS3 force field. Dynamic simulations were carried out by choosing selected docking poses from molecular docking. Detailed procedures were described by Tiwari et al. [13]. The binding free energy (MM-GBSA) was also calculated from the last 5 ns of the ligand-protein dynamic simulation trajectory [19].

## 4. Conclusions

In this study, 10 endoperoxides were selected based on previously reported results to further evaluate their cytotoxic effects against 5 different cancer cell lines and the corresponding drug-resistant cell lines. The results of the rhodamine 123 exclusion assay showed that only peroxides **7** and **8** inhibited P-gp mediated drug efflux. These results were verified by docking and molecular dynamic studies, which showed a suitable binding between **7**/**8** with P-gp.

To elucidate the mechanism of action, the most promising derivatives **7** and **8** were selected for further investigations. The results showed that endoperoxide-induced apoptosis was associated with the hyperpolarization of cell membrane potential, but ROS and caspase-3 were not involved in the mechanistic pathway.

## Figures and Tables

**Table 1:** Cytotoxicity of compounds **1**–**10** against wild-type and drug-resistant cancer cell lines with selectivity index (S.I.), compared to paclitaxel (**PTX**) and cisplatin (**CDDP**). Selectivity index (S.I.) = IC_50_ of wild-type cells/IC_50_ of resistant cancer cells.

Cmp	A549T Cells IC_50_ (μM) (SD)	S.I. ^a^	A2780 Cells IC_50_ (μM) (SD)	A2780/CDDP Cells IC_50_ (μM) (SD)	S.I.	MCF7 Cells IC_50_^a^ (μM) (SD)	MCF7/ADR Cells IC_50_ (μM) (SD)	S.I.
**1**	4.06 ± 1.70	1.22	43.65 ± 0.35	65.81 ± 3.00	0.66	>100	85.44 ± 2.90	1.17
**2**	18.29 ± 3.12	0.28	21.05 ± 6.45	53.08 ± 5.89	0.40	>100	>100	---
**3**	2.63 ± 0.21	1.68	14.65 ± 2.77	>100	---	>100	95.50 ± 3.80	1.04
**4**	0.92 ± 0.05	5.09	5.88 ± 10.43	24.08 ± 3.06	0.24	>100	18.62 ± 0.77	5.37
**5**	1.21 ± 0.15	2.42	13.80 ± 3.09	22.90 ± 7.90	0.6	>100	84.14 ± 2.88	1.18
**6**	0.57 ± 0.01	3.63	12.11 ± 4.77	>100	---	>100	>100	---
**7**	2.93 ± 0.53	0.24	1.50 ± 1.50	14.18 ± 1.22	0.15	65.51 ± 3.06	12.50 ± 4.30	5.24
**8**	11.84 ± 0.28	0.07	2.86 ± 3.30	4.95 ± 3.40	0.58	69.71 ± 3.06	22.26 ± 2.80	3.13
**9**	1.94 ± 0.09	0.74	53.91 ± 4.77	52.4 ± 5.20	1.02	>100	68.39 ± 1.22	1.46
**10**	0.96 ± 0.15	0.41	>100	>100	---	>100	>100	---
PTX	33.24 ± 2.51	---	>100	>100	---	>100	>100	---
CDDP	6.53 ± 0.39	0.41	7.56 ± 1.75	60.72 ± 3.06	0.12	>100	28.84 ± 2.88	3.64

^a^ S.I. was calculated by IC_50_ values of A549/WT cells retrieved from the work of [13].

## Data Availability

Data are contained within the article and Supplementary Materials.

## Supplementary Materials

The following are available online at https://www.mdpi.com/article/10.3390/ph15030360/s1, Figures S1–S10: Cytotoxicity assay; Figures S11–S18: Rhodamine 123 exclusion assay of **1**–**10**; Figure S19: UV-Vis analysis of oxidation of reduced riboflavin (RFH2) by trioxane **8**; Figure S20: Validation of docking reliability by using the known crystallize X-ray structure of target protein P-gp complexed with Zosuquidar; Table S1: Cytotoxicity studies [37]; Table S2: Docking scores of synthesized compounds **7** and **8** against P-gp protein; Table S3: MM-GBSA result for **7**, **8** and artesunic acid.
