# Supplementary material for: Biological Evaluation in Resistant Cancer Cells and Study of Mechanism of Action of Arylvinyl-1,2,4-Trioxanes
Source: Pharmaceuticals (Basel). 2022 Mar 16;15(3):360. doi: 10.3390/ph15030360 (PMC8955836; doi:10.3390/ph15030360)
Supplement: Supplementary file 1 [file pharmaceuticals-15-00360-s001.zip › pharmaceuticals-1588880-supplementary.pdf]

Biological evaluation in resistant cancer cells and study of mechanism of action of Arylvinyl-1,2,4-Trioxanes

Jerome P. L. Ng [1,†], Mohit K. Tiwari [2,†], Ali Adnan Nasim [1], Rui Long Zhang [1], Yuanqing Qu [1], Richa Sharma [2], Betty Yuen Kwan Law [1], Dharmendra K. Yadav [3,*] Sandeep Chaudhary [2,4,*], Paolo Coghi [5,*] and Vincent Kam Wai Wong [1,*]

1. Neher's Biophysics Laboratory for Innovative Drug Discovery, State Key Laboratory of Quality Research in Chinese Medicine, Macau University of Science and Technology, Macau 999078, China; plng@must.edu.mo (J.P.L.N.); aanasim@gmail.com (A.A.N.); yeyutingfengmo@gmail.com (R.L.Z.); brookqu@gmail.com (Y.Q.).; yklaw@must.edu.mo (B.Y.K.L.); bowaiwong@gmail.com (V.K.W.W.)
2. Laboratory of Organic and Medicinal Chemistry, Department of Chemistry, Malaviya National Institute of Technology, Jawaharlal Nehru Marg, Jaipur 302017, India; tiwari.mohitpharma22@gmail.com (M.T.); richasharmachem1@gmail.com (R.S.); schaudhary.chy@mnit.ac.in (S.C.);
3. College of Pharmacy, Gachon University of Medicine and Science, Incheon City 21924, Korea; dharmendra30oct@gmail.com (D.K.Y.);
4. Laboratory of Organic and Medicinal Chemistry (OMC lab) National Institute of Pharmaceutical Education and Research (NIPER-R) Raebareli, Lucknow 226002, India; schaudhary.chy@niperraebareli.edu.in (S.C.);
5. School of Pharmacy, Macau University of Science and Technology, Macau, China; coghips@must.edu.mo (P.C.).

*Correspondence: dharmendra30oct@gmail.com (D.K. Y.); schaudhary.chy@mnit.ac.in (S.C.); coghips@must.edu.mo (P.C.); bowaiwong@gmail.com (V.K.W.W.)
†Both authors have equal contribution.

Supplementary Materials

Table S1. Additional cell lines data **4**

Cytotoxicity Assays **5**

Figure S1 Cytotoxicity assay against A549T cell lines *5*

Figure S2 Cytotoxicity assay against A2780/WT cell lines *6*

Figure S3 Cytotoxicity assay against A2780/CDDP cell lines *7*

Figure S4. Cytotoxicity assay against HCT-8/WT cell lines *8*

Figure S5. Cytotoxicity assay against HCT-8 TR cell lines *9*

Figure S6. Cytotoxicity assay against MCF-7 WT cell lines *10*

Figure S7. Cytotoxicity assay against MCF-7/ADR cell lines *11*

Figure S8. Cytotoxicity assay against SGC7901 WT cell lines *12*

Figure S9. Cytotoxicity assay against SGC7901/CDDPR cell lines *13*

Figure S10. Cytotoxicity assay against LO2 and CCD19Lu normal cell lines *13*

Rhodamine 123 Exclusion Assays **14**

Figure S11. Compound ***1*** *14*

Figure S12. Compound ***2*** *14*

Figure S13. Compound ***3*** *15*

Figure S14. Compound ***4*** *15*

Figure S15. Compound ***5*** *16*

Figure S16. Compound ***6*** *16*

Figure S17. Compound ***9*** *16*

Figure S18. Compound ***10*** *16*

UV-Vis analysis **19**

Figure S19. UV-Vis analysis of oxidation of reduced riboflavin (RFH2) by trioxane 8. .... ***Error! Bookmark not defined.***

In silico **studies** **20**

Figure S20. Validation of docking reliability by using the known crystallize X-ray structure of target protein P-gp complexed with Zosuquidar *20*

Table S2. Docking scores of synthesized and standard compound ***7*** *and* ***8*** *against P-gp protein (PDB ID: 6FN1)* 20

Table S3. MM-GBSA result for ***7****,* ***8*** *and Artesunic acid* 21

References 21

Cytotoxicity studies

Table S1. Additional data of cell lines

Cytotoxicity of compounds **1-10** against normal, wild-type cancer and resistant cancer cell lines with selectivity index (S.I), compared to paclitaxel (**PTX**) and Cisplatin (**CDDP**).

Selectivity index (S.I.) = IC_{50} for wild type cells / IC_{50} for resistant cancer cells

HCT-8/TR: Taxol-resistant human colorectal cancer cell line

SGC7901/CDDP: Cisplatin-resistant human gastric cancer cell line

Compounds	LO2 cells IC_{50}[μM] (SD)	CCD-19Lu IC_{50}[μM] (SD)	HCT-8/WT cells IC_{50}[μM] (SD)	HCT-8/TR cells IC_{50}[μM] (SD)	S.I.	SGC7901 cells IC_{50}[μM] (SD)	SGC7901/CDDP cells IC_{50} [μM] (SD)	S.I.
**1**	-	-	41.052±2.1	55.16±4.4	0.74	>100	>100	---
**2**	-	-	52.28±3.2	58.43±5.3	0.89	>100	>100	---
**3**	-	-	51.48±2.77	39.05±3.2	1.32	>100	>100	---
**4**	-	-	17.44±5.2	19.20±4.2	0.9	68.39±2.2	28.73±0.1	2.38
**5**	-	-	50±3.9	38.16±3.60	1.31	>100	>100	---
**6**	-	-	>100	>100	---	>100	>100	---
**7**	5.27±0.03	2.36±0.20	12.20±1.22	3.51±0.2	3.47	7.82±1.2	7.44±1.1	1.05
**8**	4.31±0.05	0.56±0.01	27.75±3.08	7.64±0.77	3.63	20.10±2.1	18.69±1.02	1.07
**9**	-	-	>100	28.76±1.08	3.48	58.43±0.1	NA	NA
**10**	-	-	>100	>100	---	>100	NA	NA
**PTX**	-	-	58.29±6.2	>100	0.58	0.17±0.1[b]	NA	NA
**CDDP**	-	-	>100	49.35±2.2	2.04	10.19±0.2	13.54±1.2	0.75

[a]Data retrieved from [1]; NA = Not applicable.

IC_{50} values for BEAS-2B were available in our previous publication [2].

Cytotoxicity Assays

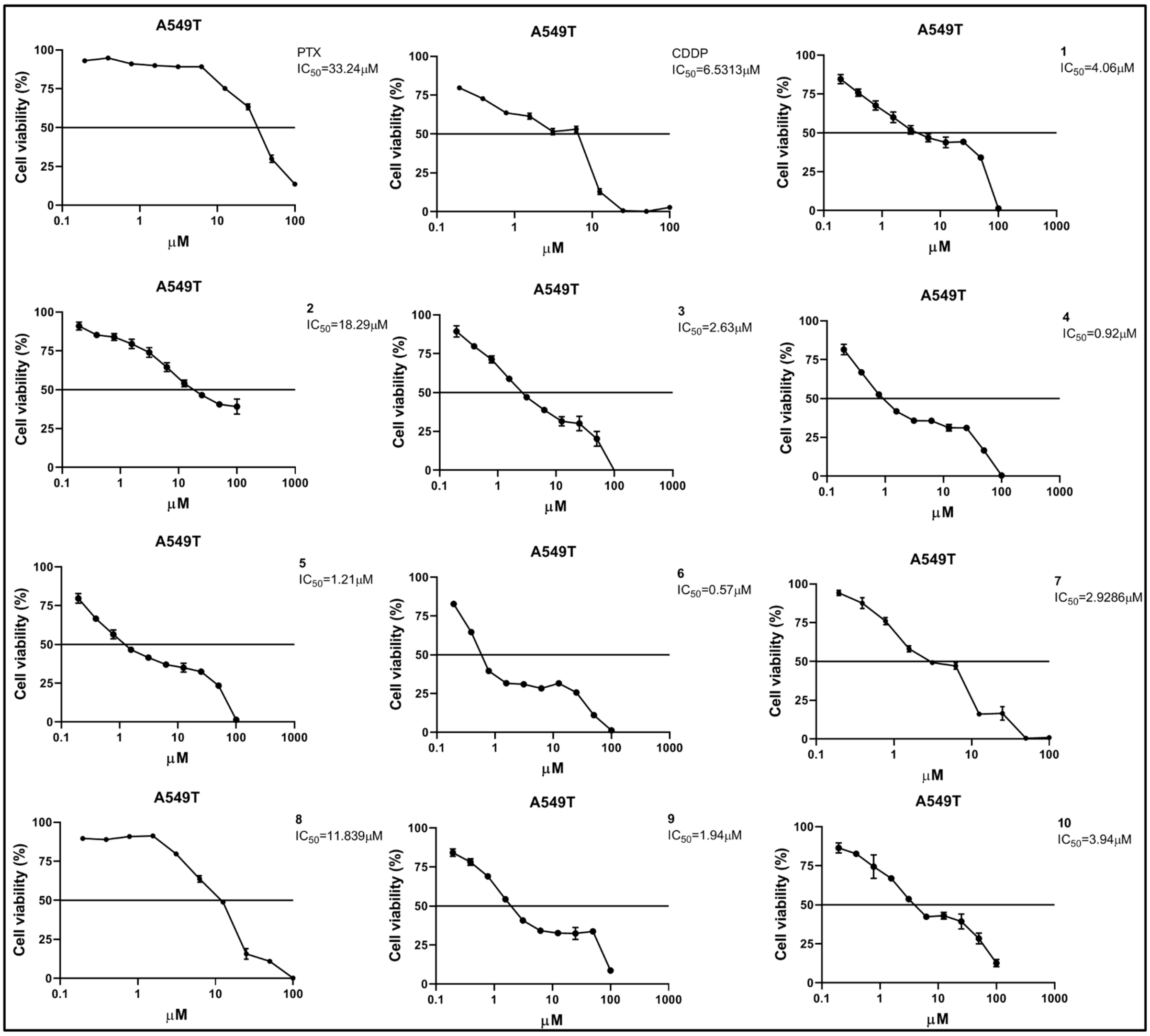

Figure S1 Cytotoxicity assay against A549T cell lines

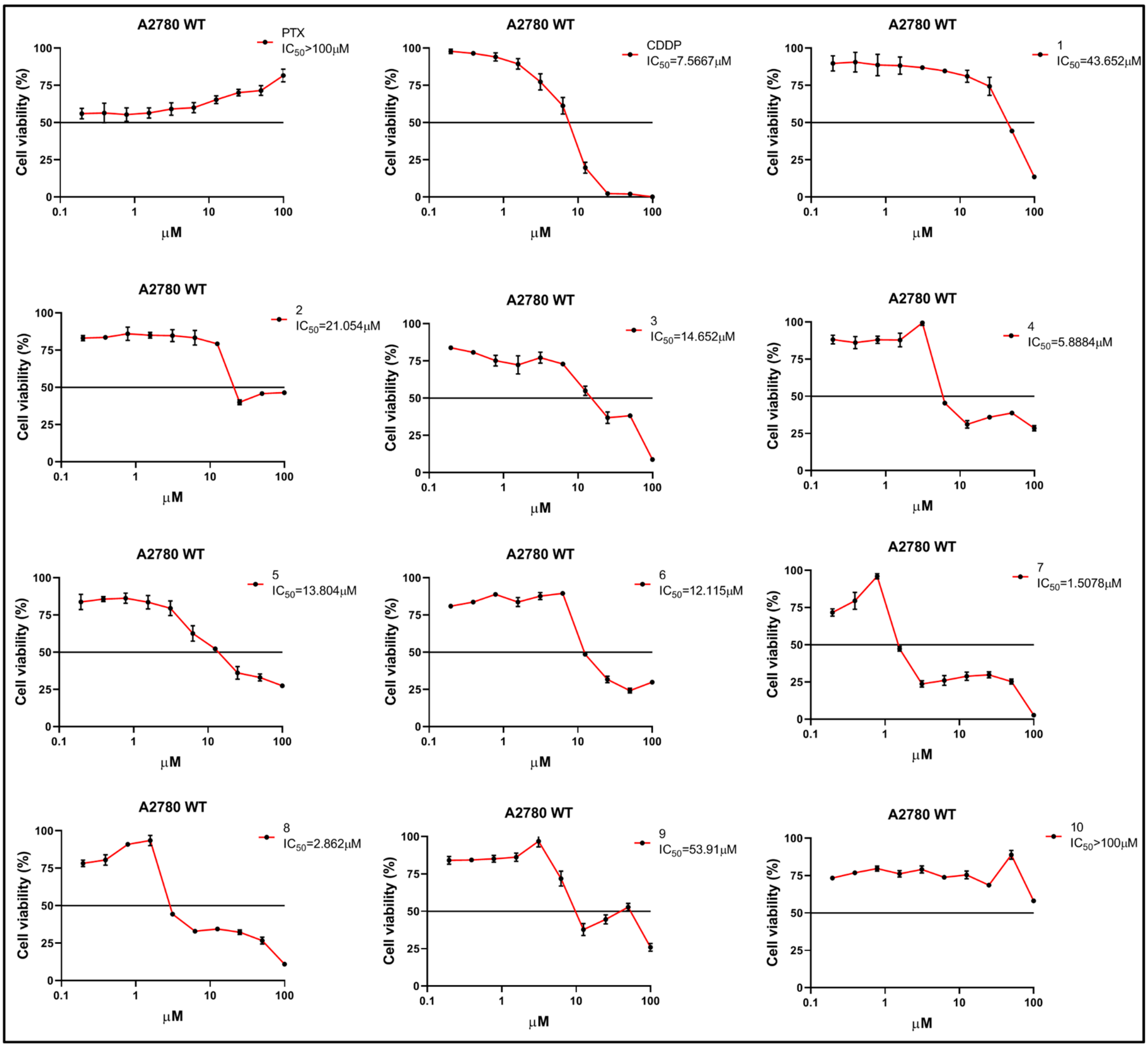

Figure S2 Cytotoxicity assay against A2780/WT cell lines

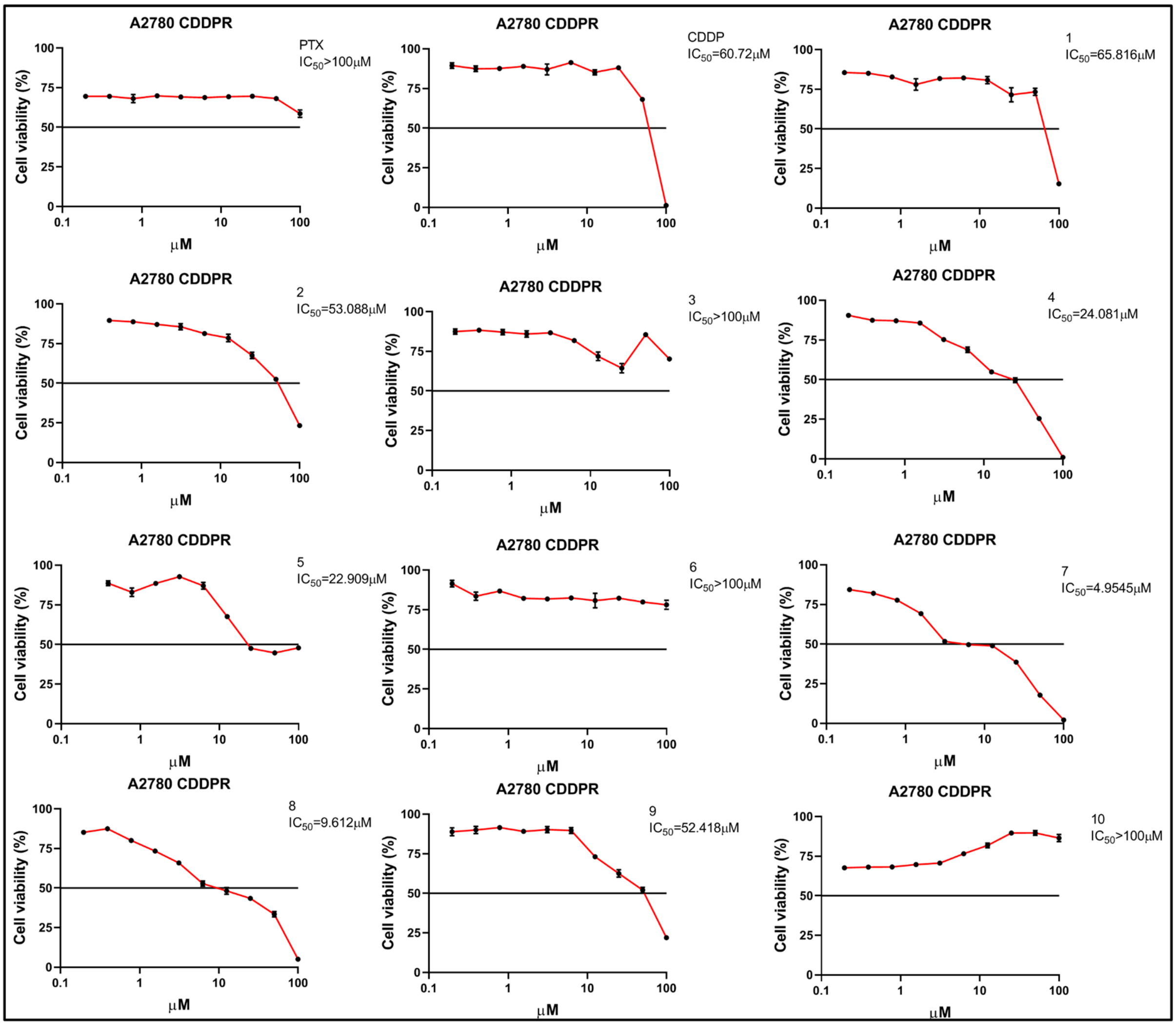

Figure S3 Cytotoxicity assay against A2780/CDDP cell lines

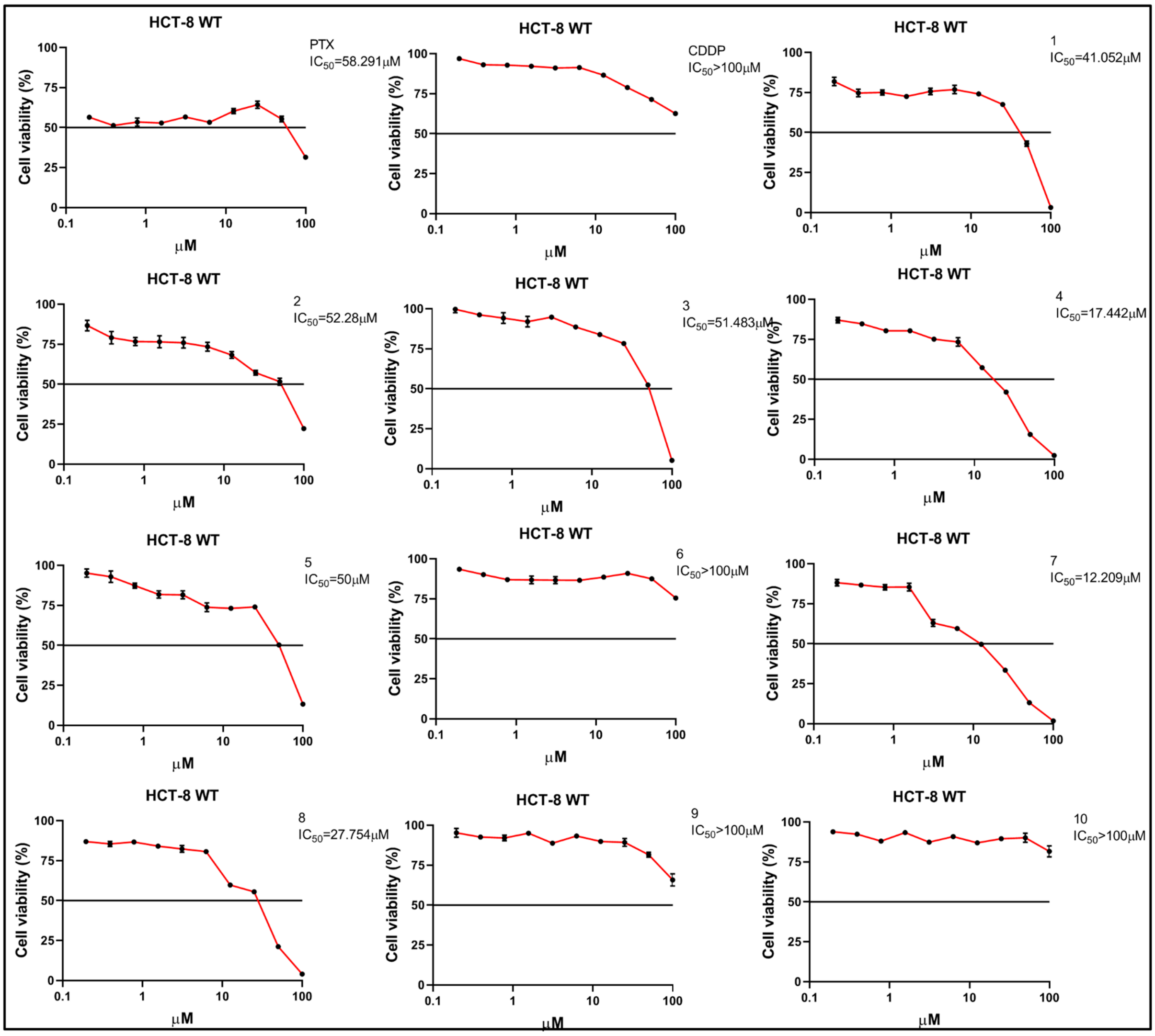

Figure S4. Cytotoxicity assay against HCT-8/WT cell lines

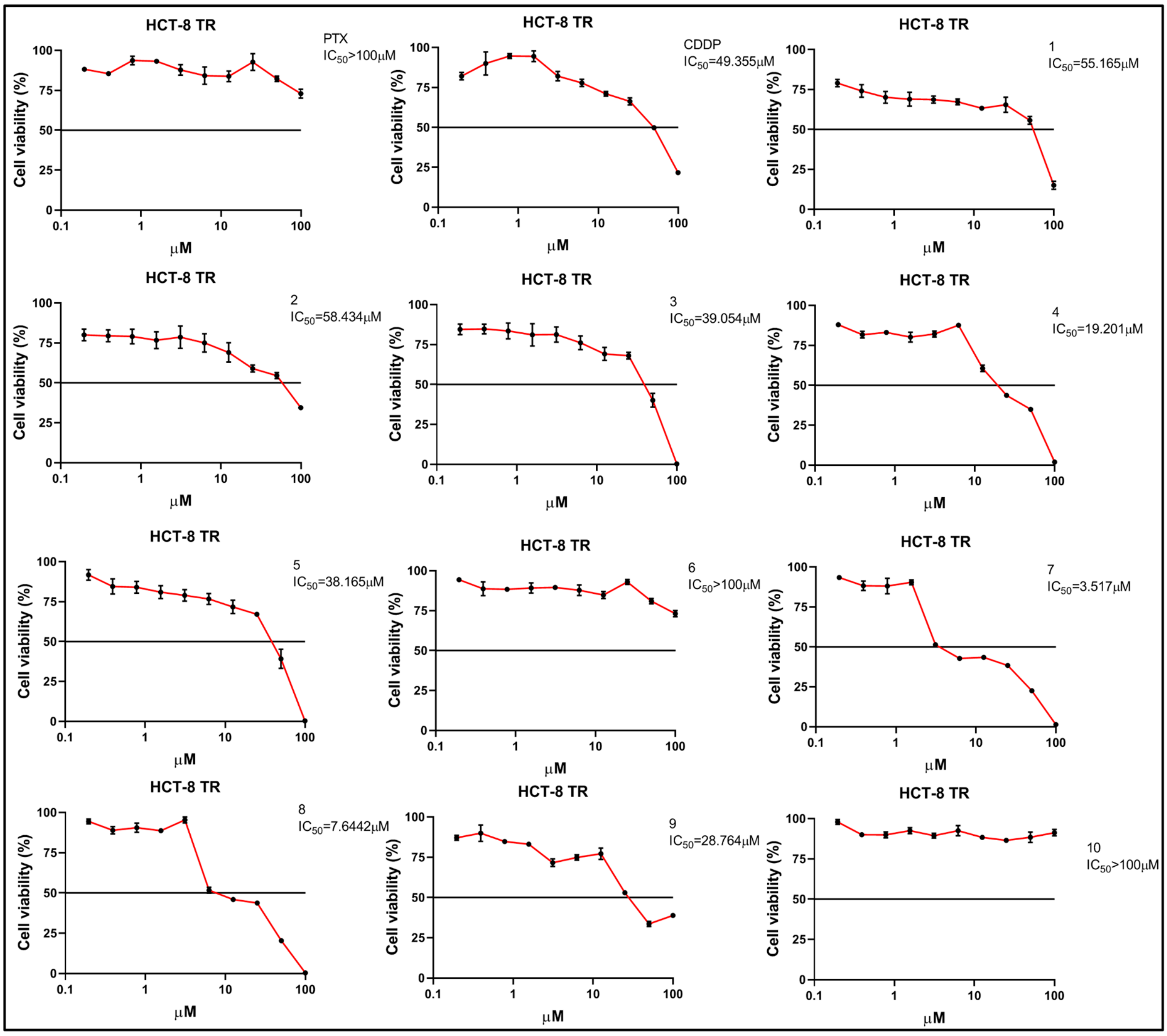

Figure S5. Cytotoxicity assay against HCT-8/TR cell lines

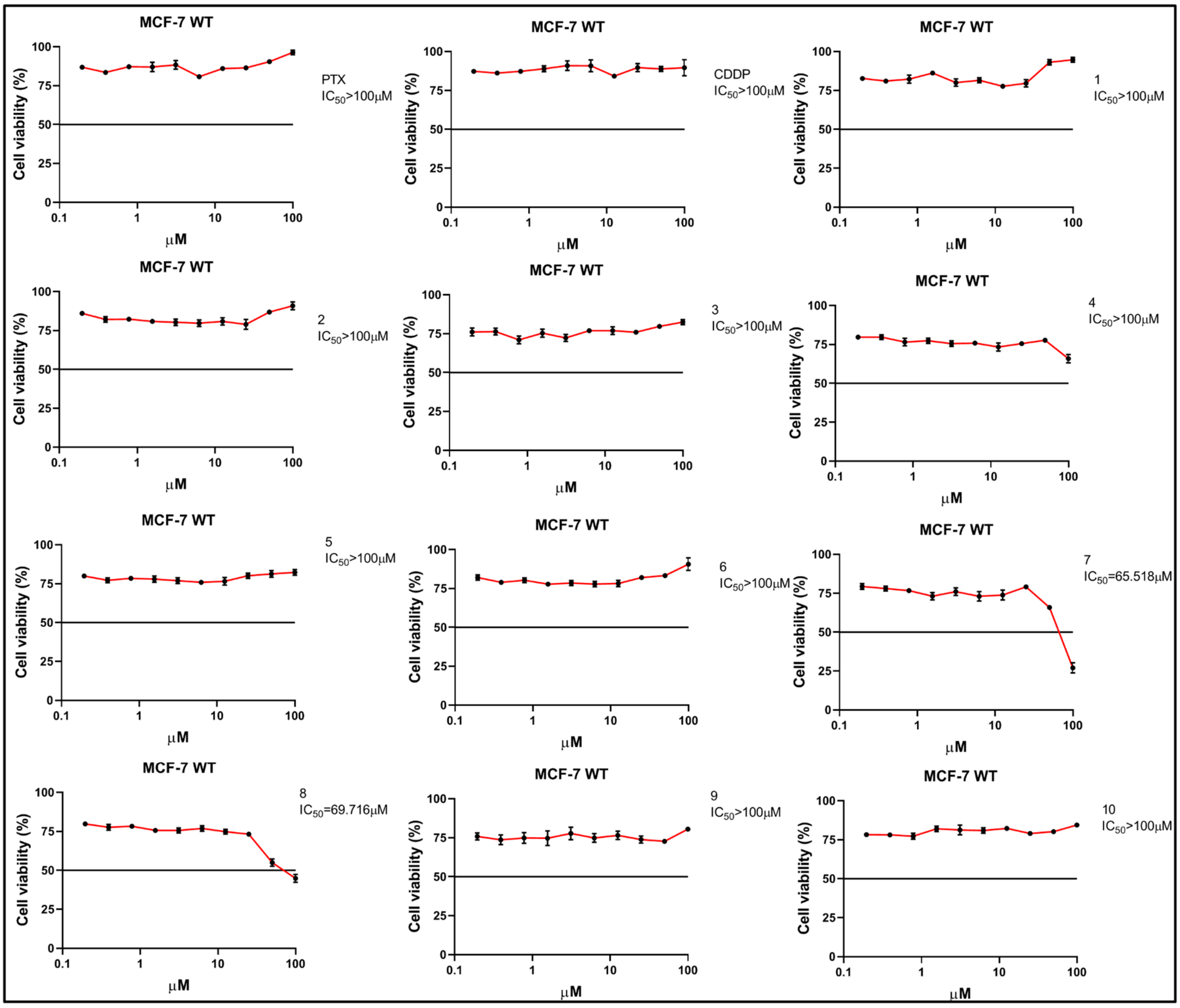

Figure S6. Cytotoxicity assay against MCF-7/WT cell lines

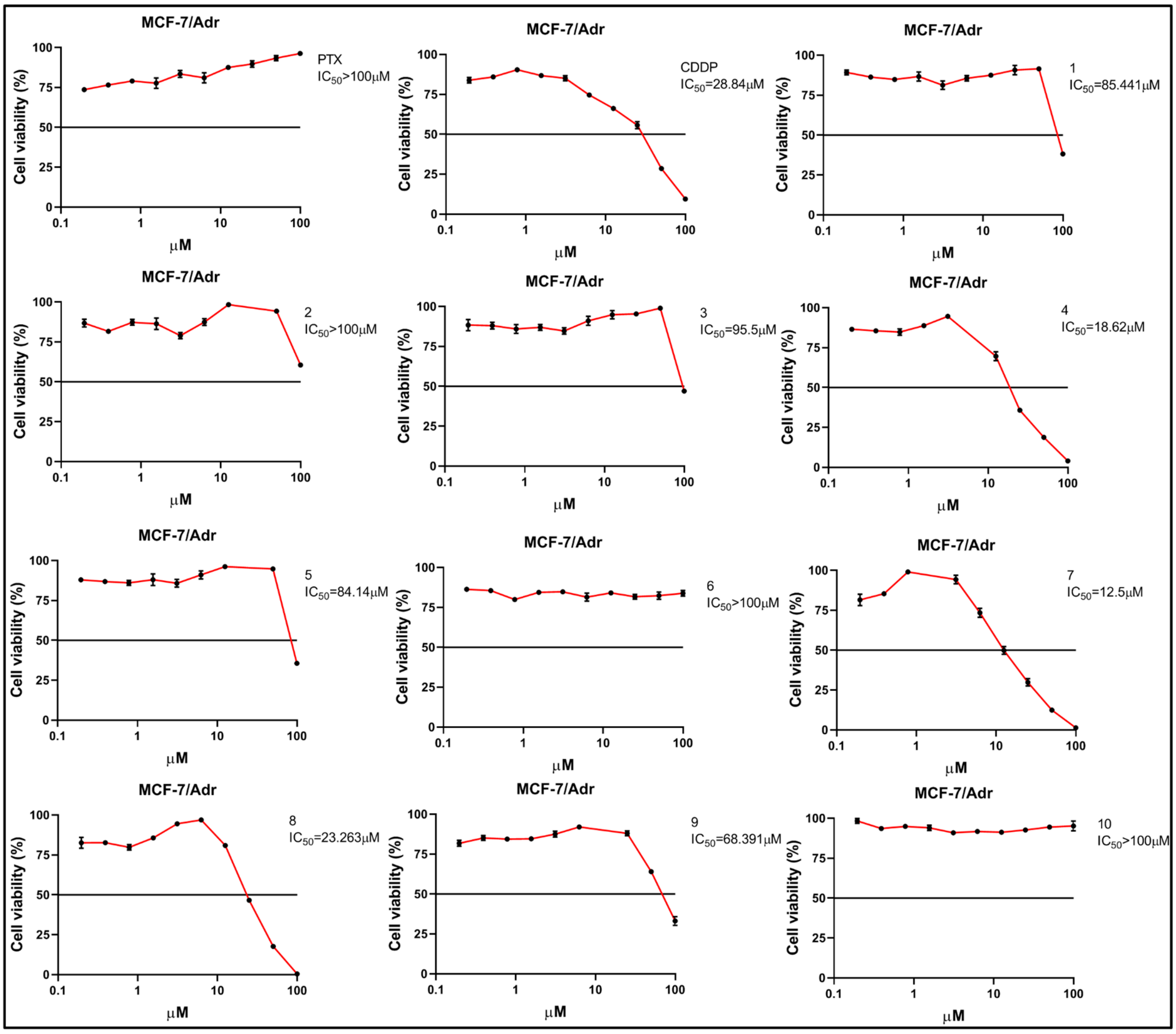

Figure S7. Cytotoxicity assay against MCF-7/ADR cell lines

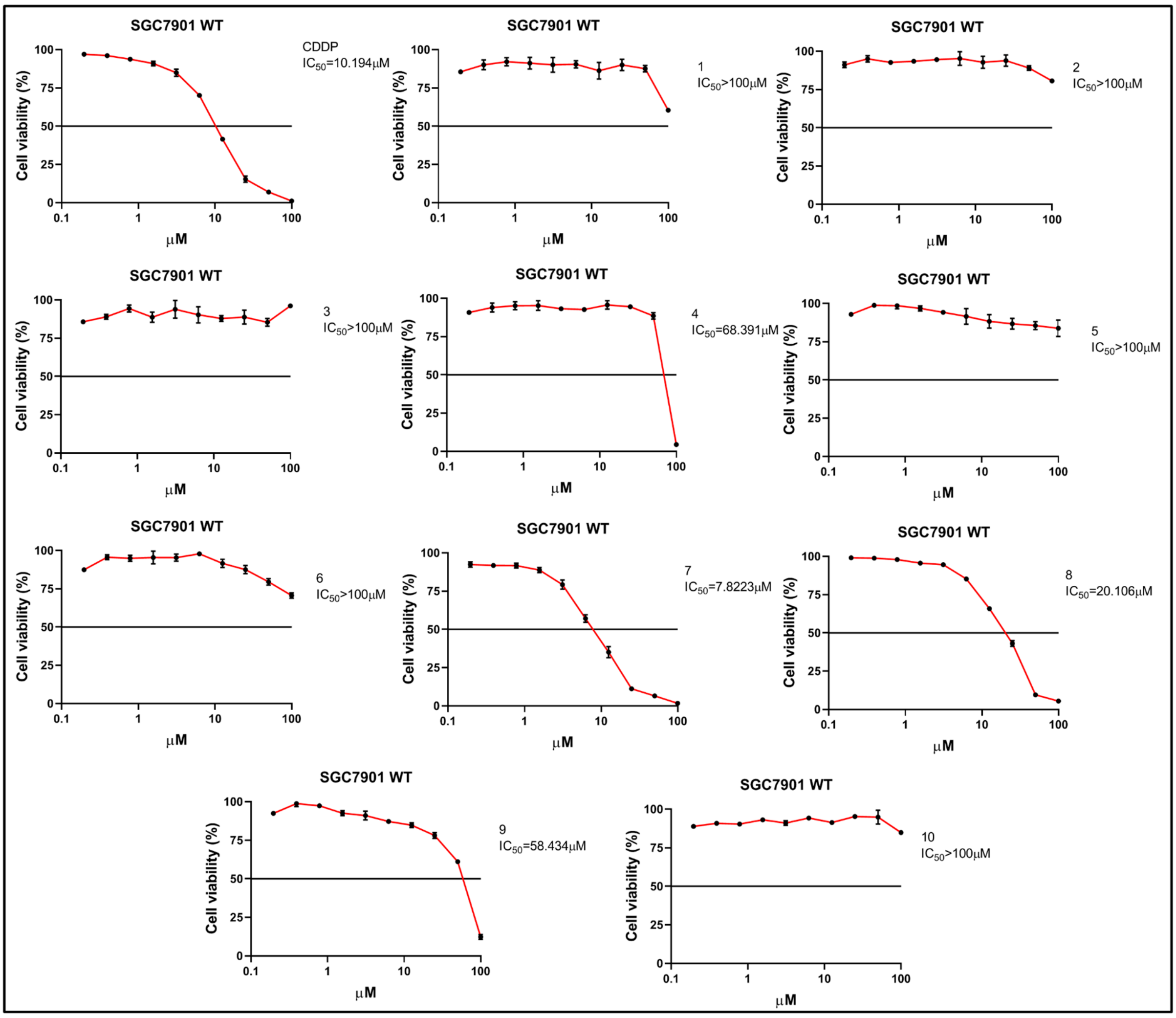

Figure S8. Cytotoxicity assay against SGC7901/WT cell lines

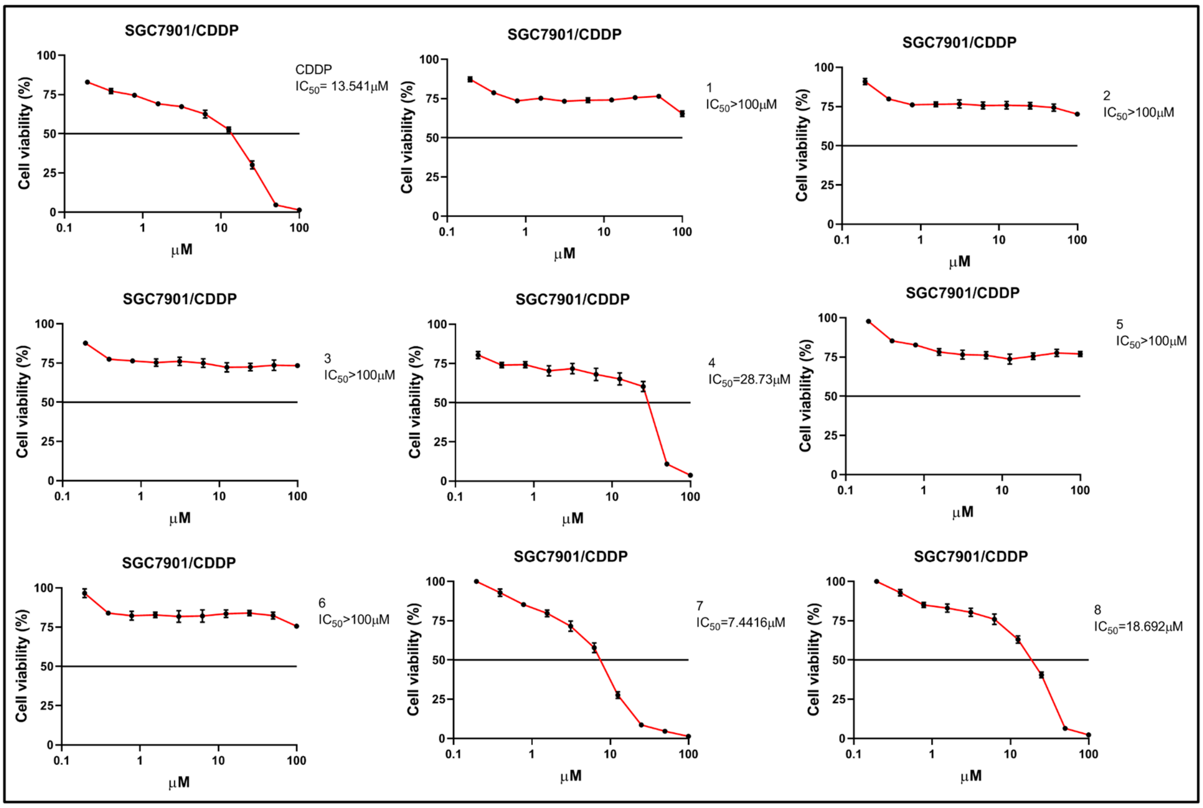

Figure S9. Cytotoxicity assay against SGC7901/CDDP cell lines

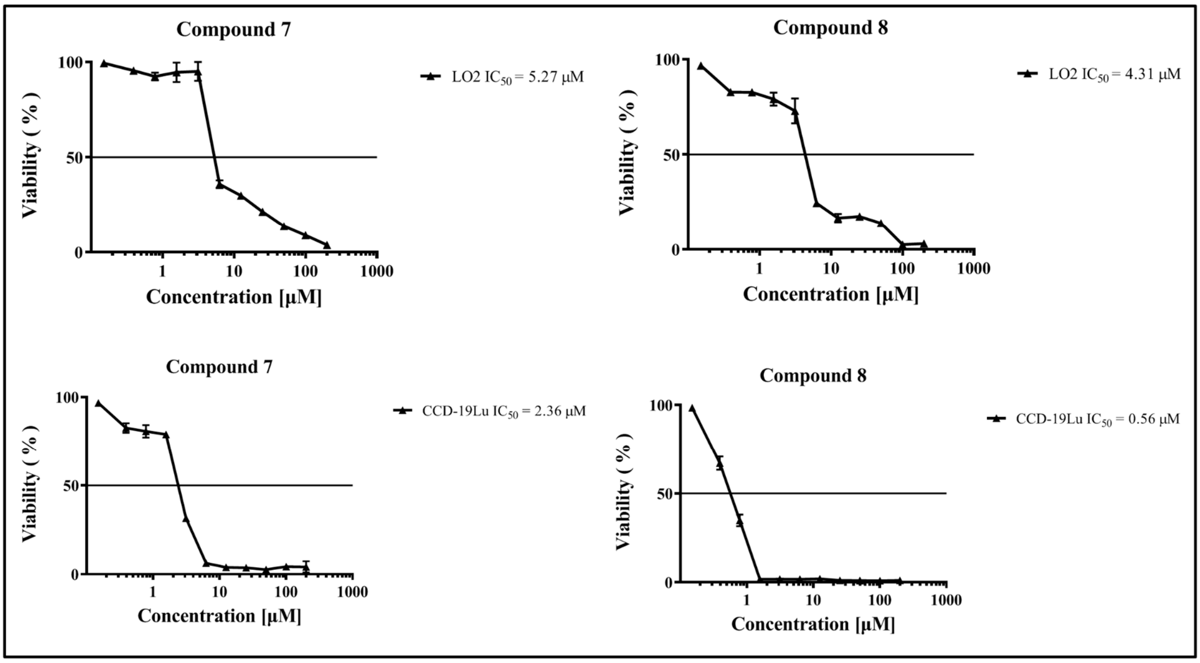

Figure S10. Cytotoxicity assay against LO2 and CCD19Lu normal cell lines

Rhodamine 123 Exclusion Assays

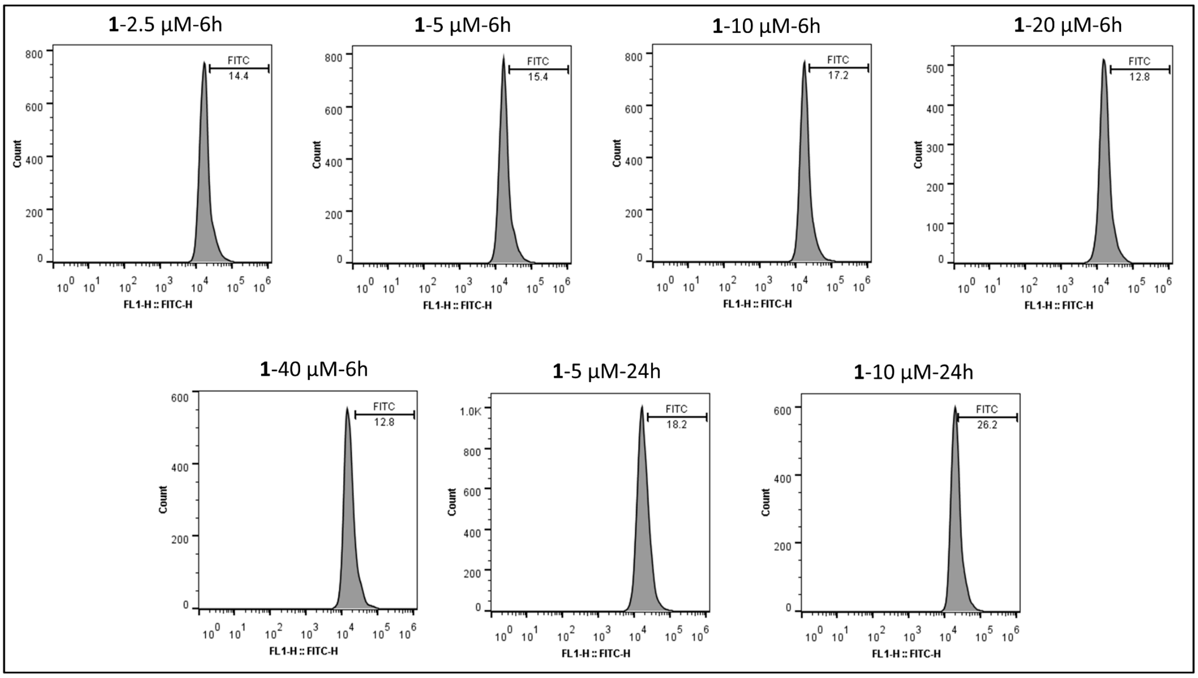

Figure S11. Compound **1**

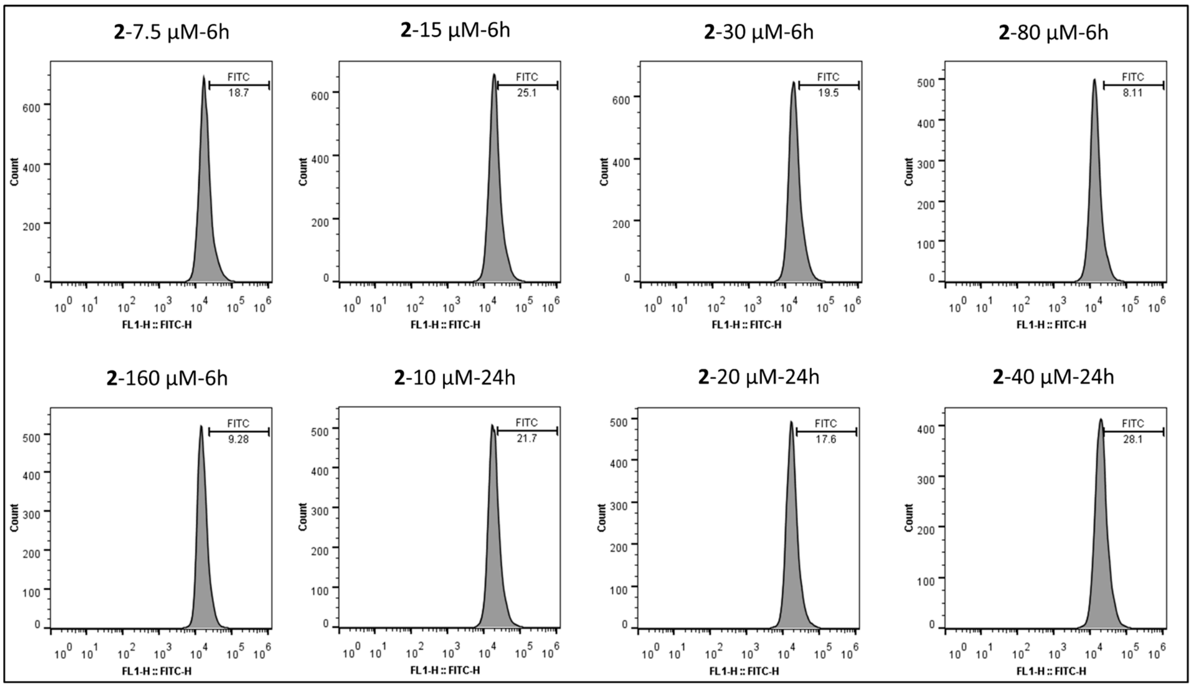

Figure S12. Compound **2**

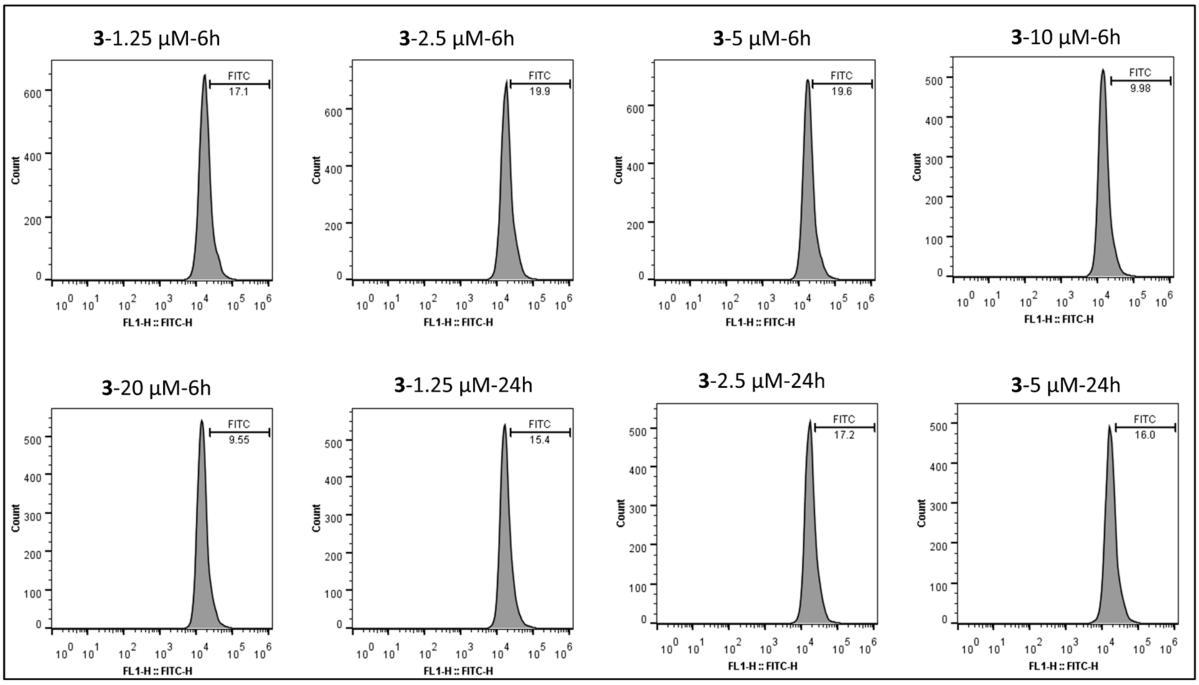

Figure S13. Compound **3**

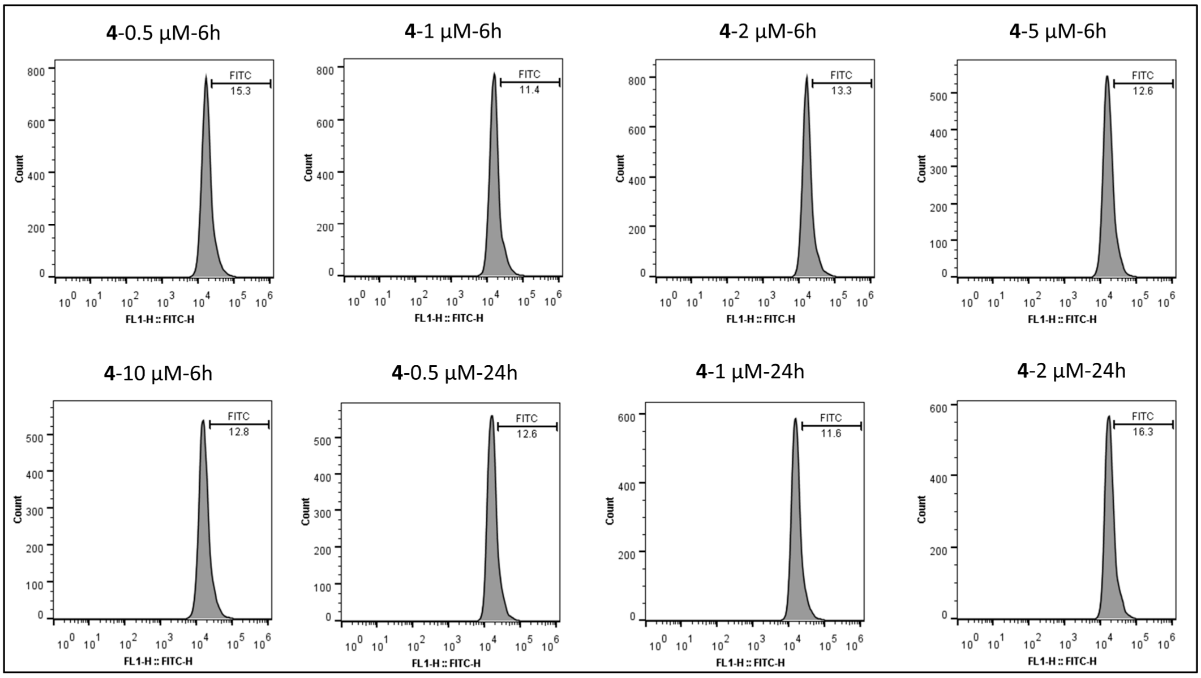

Figure S14. Compound **4**

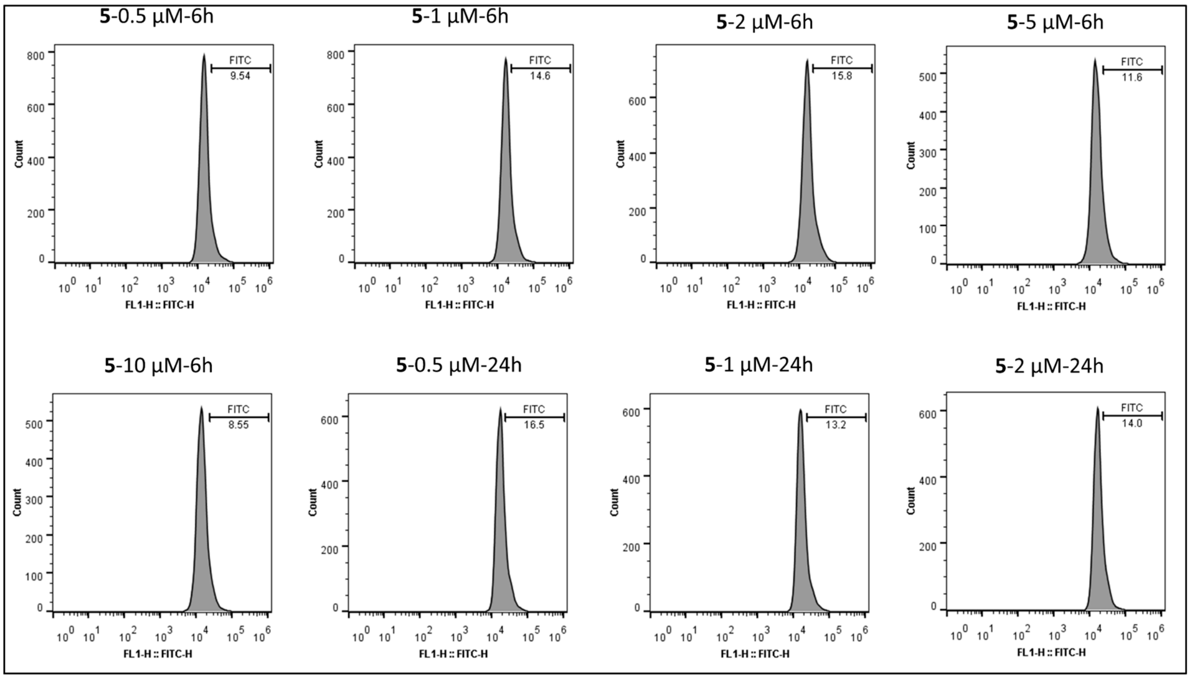

Figure S15. Compound **5**

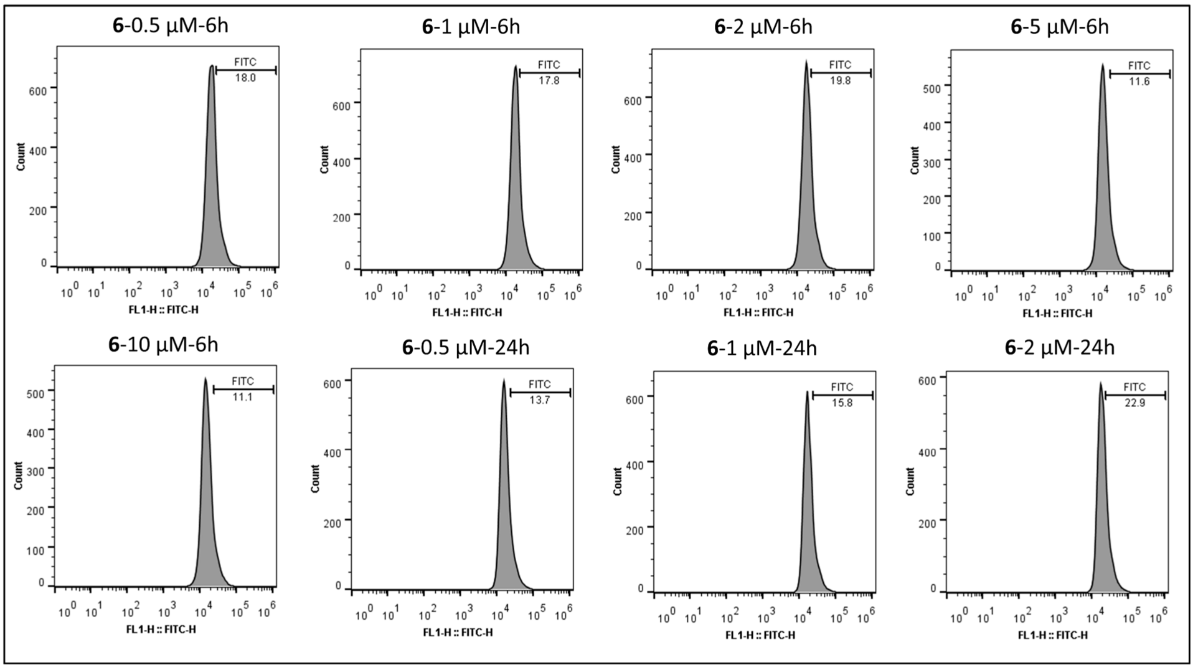

Figure S16. Compound **6**

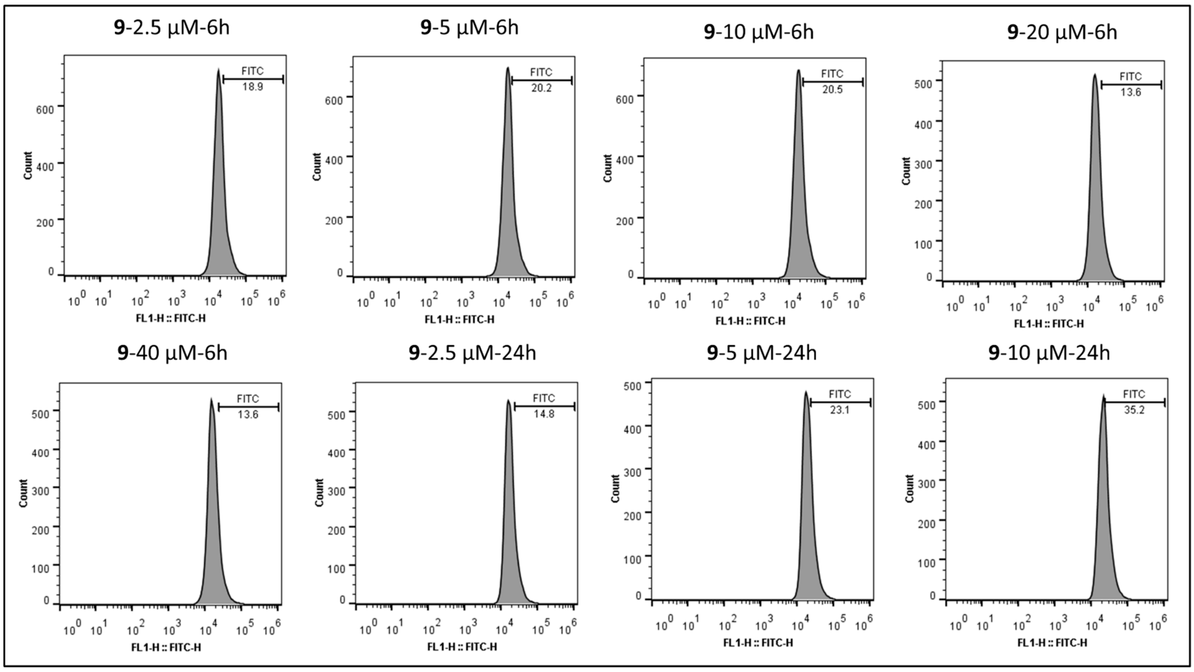

Figure S17. Compound 9

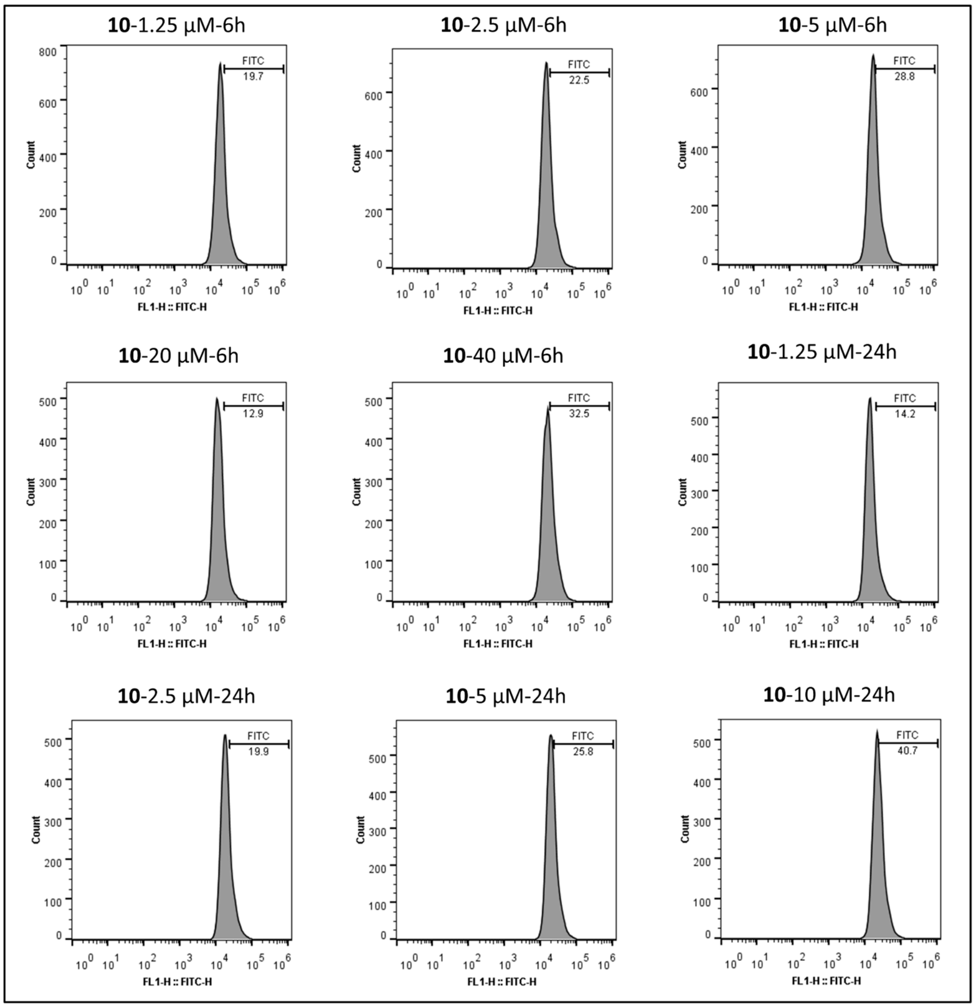

Figure S18. Compound 10

.

UV-Vis analysis

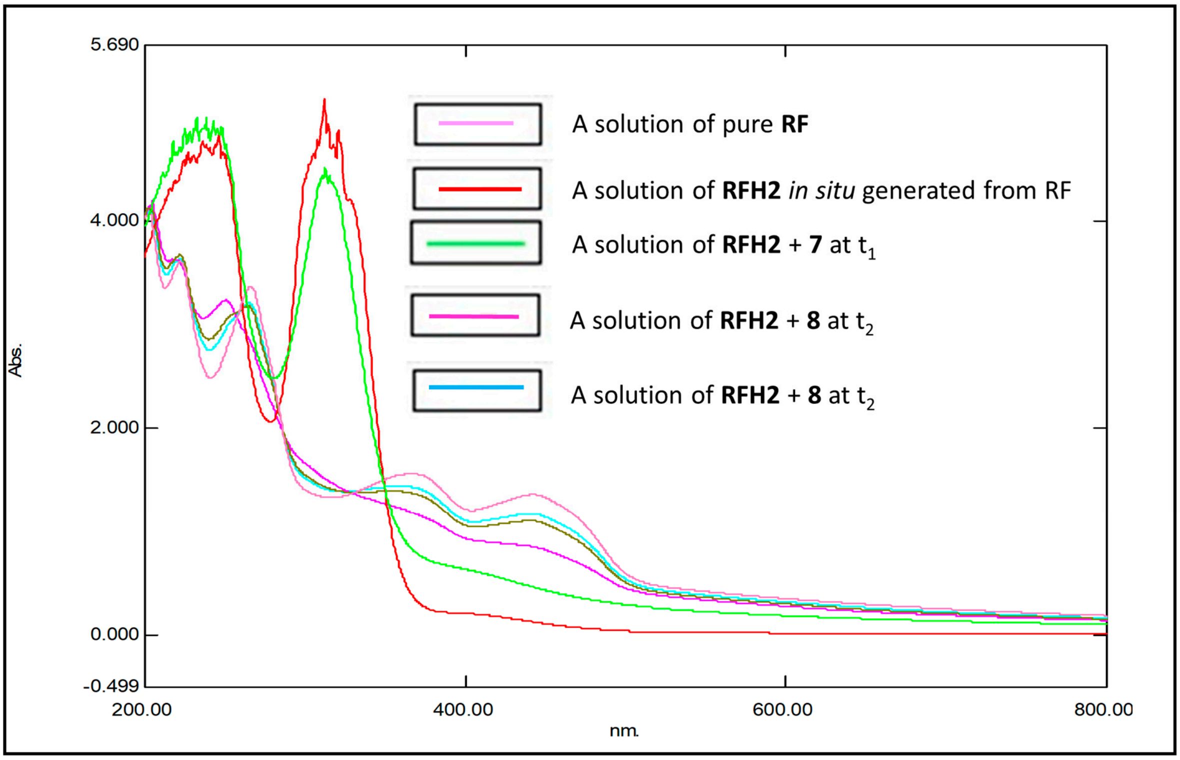

Figure S19 UV-Vis analysis of oxidation of reduced riboflavin (RFH2) by trioxane **8**. RF (6.6 x 10^{-4} mmol) was completely converted to RFH2 (disappearance of absorption bands at 370 and 445 nm) by the treatment with sodium dithionite (1.4 x 10^{-4} mmol) under argon at 37 °C. Trioxane **8** (4 x 10^{-4} mmol) in MeCN was added, and the absorptions of the mixture at 370 and 445 nm were monitored at an interval (t_1-t_3) of 20 s until complete oxidation of RFH2 back to RF (within 10 min).

In silico studies

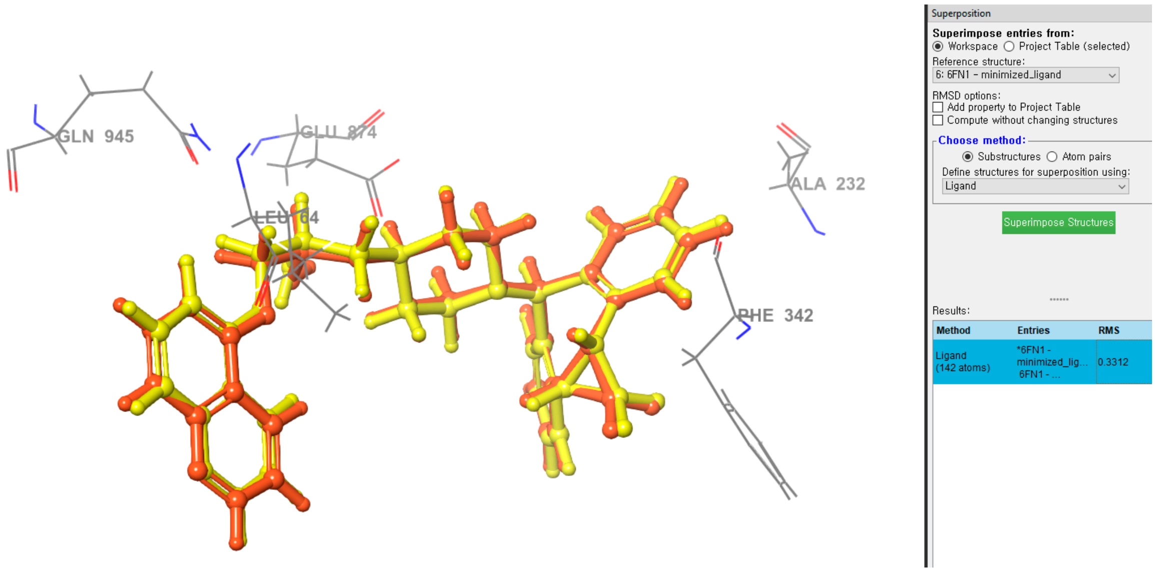

Figure S20. Validation of docking reliability by using the known crystallize X-ray structure of target protein P-gp complexed with Zosuquidar.

The docking reliability was validated by using the known X-ray crystal structure of target protein P-gp complexed with Zosuquidar. The co-crystallized Zosuquidar inhibitor was redocked into the binding site, and the docked conformation with the highest score of -7.479 was selected as the most probable binding conformation. The low root mean-square deviation (RMSD) of 0.3312 Å between the docked and the crystal conformations indicates the high reliability of Glide software in reproducing the experimentally observed binding mode for this inhibitor. As shown in Figure S20, redocked molecules were almost in the same position with co-crystallized at the active site of Zosuquidar inhibitor.

Table S2. Docking scores of synthesized and standard compound **7** and **8** against P-gp protein (PDB ID: 6FN1)

Entry	Compound	Docking score (kcal/mol)	Glide XP Energy (kcal/mol)	Glide XP Emodel (kcal/mol)
1	**7**	-7.089	-7.089	-42.051
2	**8**	-8.196	-8.196	-36.935
3	**Artesunic acid**	-6.252	-6.253	-36.817

Table S3:MM-GBSA result for **7**, **8** and Artesunic acid

Entry	dG (Bind)	dG (Coulomb)	dG (Covalent)	dG (Hbond)	dG (Lipo)	dG (Solv_GB)	dG (vdW)
**7**	-96.714	-5.118	0.032	-0.010	-53.757	10.733	-45.193
**8**	-93.841	-3.696	0.766	-0.10	-54.440	10.042	-46.006
**Artesunic acid**	-70.435	-13.595	1.111	-0.027	-39.027	25.087	-43.984

References

1. Shang, C.; Sun, L.; Zhang, J.; Zhao, B.; Chen, X.; Xu, H.; Huang, B. Silence of cancer susceptibility candidate 9 inhibits gastric cancer and reverses chemoresistance. *Oncotarget*. **2017**, *8*, 15393-15398, doi: 10.18632/oncotarget.14871.
2. Tiwari, M. K.; Coghi, P.; Agrawal, P.; Yadav, D. K.; Yang, L. J.; Congling, Q.; Sahal, D.; Wong, V. K. W.; Chaudhary, S. Novel Halogenated Arylvinyl-1,2,4 Trioxanes as Potent Antiplasmodial as well as Anticancer Agents: Synthesis, Bioevaluation, Structure-Activity Relationship and In-silico Studies. *Eur. J. Med. Chem.* **2021**, *224*, 113685.
